# Structural Basis of the Subcellular Topology Landscape of *Escherichia coli*

**DOI:** 10.3389/fmicb.2019.01670

**Published:** 2019-07-24

**Authors:** Maria S. Loos, Reshmi Ramakrishnan, Wim Vranken, Alexandra Tsirigotaki, Evrydiki-Pandora Tsare, Valentina Zorzini, Jozefien De Geyter, Biao Yuan, Ioannis Tsamardinos, Maria Klappa, Joost Schymkowitz, Frederic Rousseau, Spyridoula Karamanou, Anastassios Economou

**Affiliations:** ^1^Department of Microbiology and Immunology, Laboratory of Molecular Bacteriology, Rega Institute, KU Leuven, Leuven, Belgium; ^2^VIB Switch Laboratory, Department for Cellular and Molecular Medicine, VIB-KU Leuven Center for Brain & Disease Research, KU Leuven, Leuven, Belgium; ^3^Interuniversity Institute of Bioinformatics in Brussels, Free University of Brussels, Brussels, Belgium; ^4^Structural Biology Brussels, Vrije Universiteit Brussel and Center for Structural Biology, Brussels, Belgium; ^5^Metabolic Engineering & Systems Biology Laboratory, Institute of Chemical Engineering Sciences, Foundation for Research and Technology-Hellas, Patras, Greece; ^6^Gnosis Data Analysis PC, Heraklion, Greece; ^7^Department of Computer Science, University of Crete, Heraklion, Greece

**Keywords:** protein secretion, cytoplasmome, protein disorder, protein domains, protein folding, protein subcellular localization, protein targeting, secretome

## Abstract

Cellular proteomes are distributed in multiple compartments: on DNA, ribosomes, on and inside membranes, or they become secreted. Structural properties that allow polypeptides to occupy subcellular niches, particularly to after crossing membranes, remain unclear. We compared intrinsic and extrinsic features in cytoplasmic and secreted polypeptides of the *Escherichia coli* K-12 proteome. Structural features between the cytoplasmome and secretome are sharply distinct, such that a signal peptide-agnostic machine learning tool distinguishes cytoplasmic from secreted proteins with 95.5% success. Cytoplasmic polypeptides are enriched in aliphatic, aromatic, charged and hydrophobic residues, unique folds and higher early folding propensities. Secretory polypeptides are enriched in polar/small amino acids, β folds, have higher backbone dynamics, higher disorder and contact order and are more often intrinsically disordered. These non-random distributions and experimental evidence imply that evolutionary pressure selected enhanced secretome flexibility, slow folding and looser structures, placing the secretome in a distinct protein class. These adaptations protect the secretome from premature folding during its cytoplasmic transit, optimize its lipid bilayer crossing and allowed it to acquire cell envelope specific chemistries. The latter may favor promiscuous multi-ligand binding, sensing of stress and cell envelope structure changes. In conclusion, enhanced flexibility, slow folding, looser structures and unique folds differentiate the secretome from the cytoplasmome. These findings have wide implications on the structural diversity and evolution of modern proteomes and the protein folding problem.

## Introduction

**FIGURE 1:**
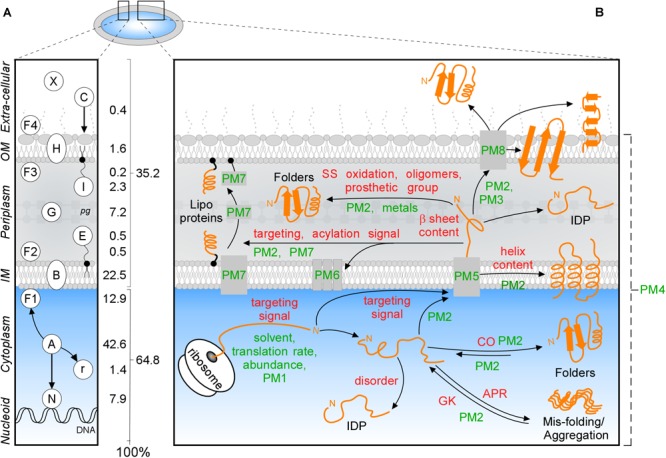
Subcellular protein distribution in K-12. **(A)** Cartoon representation of an *Escherichia coli* cell that comprises the cytoplasm surrounded by the inner (IM) and outer (OM) membranes separated by periplasm with peptidoglycan (pg). The symbols of different classes are noted as letters in the left box, the percentages of the whole K-12 proteome are in the right box. In K-12, cytoplasmic proteins include those binding to the nucleoid (N), peripheral inner membrane (F1), associated with ribosomes (r) or are freely diffusing (A); Exportome is either embedded in the IM (B) or comprises the secretome. These are proteins translocated across the IM: periplasmic enzymes (G), lipoproteins (I, E), OM-embedded proteins (H), IM and OM peripherally associated periplasmic proteins (F2, F3), surface-exposed appendages like flagella, fimbriae, and curli or are fully secreted to the extracellular space (X) and colicins (C) are imported through the cell envelope. **(B)** Intrinsic factors (red) and extrinsic (green) that bias the diffusion of a protein (orange) toward its final destination and folding. Proteostatic machineries (PM): PM1 = chaperones residing on the ribosome (SRP, SecA, TF); PM2 = soluble chaperones; PM3 = pilotin; PM4 = proteases; PM5 = IM transport channels; PM6 = lipoprotein modification module; PM7 = lipid factors; PM8 = OM insertion machineries (Bam and Omp). APR, aggregation prone region; CO, contact order; IDP, intrinsically disordered protein; GK, gatekeepers.

All cells have specialized, membrane-bound subcellular compartments. More than a third of their proteome exits the cytoplasm after synthesis. How proteins find these extra-cytoplasmic locations, enter them after crossing membranes and acquire folded states, is a central biological problem. Gram^-^ bacterial cells, like the *Escherichia coli* K-12 model, have a cytoplasm bound by a multi-layered cell envelope consisting of: the IM phospholipid bilayer; the periplasm (containing proteins, small molecules and the peptidoglycan mesh); an additional external lipid bilayer and the OM, which also contains anchored lipopolysaccharide molecules (Figure 1A; Silhavy et al., 2010).

Polypeptides that exit the cytoplasm of *E. coli*, i.e., the “exportome,” are either embedded in the IM (IM proteins or “membranome”) or comprise the “secretome.” Secreted proteins reside in the cell envelope or become fully released in the surrounding milieu (Figure 1A). The exportome is involved in many cellular processes such as membrane biogenesis, cell structure maintenance, transport and signaling. Several proteins undergo dynamic location changes, e.g., nucleoid to membrane or cytoplasm to extracellular space. Understanding protein subcellular locations, interactions and dynamics is important for the physicochemical understanding and the *in silico* modeling of cells, their evolutionary connections, environmental responses, pathologies, chemotherapeutic interventions and biotechnological re-engineering.

Protein trafficking overcomes multiple challenges: recognition and sorting of protein “leavers” from “remainers” in the crowded cytoplasm, association with and crossing of or entry into membranes and protein folding that is delayed, until final destinations are reached. Trafficking is influenced by several extensively studied (De Geyter et al., 2016) environmental extrinsic factors, like the environment that the nascent polypeptide encounters, protein concentrations, proteostatic machineries and translocases (Figure 1B, green). In addition, polypeptides have their own, poorly understood, intrinsic physicochemical properties. These include signal peptides, disordered regions or specific 3D folds such as TM helices (Lemmin et al., 2013), β-barrels (Wimley, 2003), amphiphilic α-helical anchors (Figure 1B, red; Parlitz et al., 2007; Sung et al., 2009) and peptidoglycan (Hizukuri et al., 2009) and DNA- (Ishihama, 2012) binding domains.

We previously curated and annotated the subcellular localization of the complete K-12 proteome (Orfanoudaki and Economou, 2014), and have updated it here. Moreover, we examined whether a protein structural basis underlies the cytoplasmome-exportome divide. We investigated more than a hundred different protein characteristics, including physicochemical and structural information, and identified multiple differences between cytoplasmic, IM and secreted proteins. While, structural differences were largely expected and known for many IM protein features, the differences between soluble cytoplasmic and secretome polypeptides, and transiently soluble OM proteins were remarkable and unexpected. Here, we describe the differences between the different topology groups in *E. coli* K-12. To our knowledge, this is the first such comprehensive study. This information is openly accessible through a database that contains all the manually curated information on *E. coli* protein topology.

Secretome intrinsic properties go well beyond the presence of signal peptides known to be required for export (Tsirigotaki et al., 2017). Secretome mature domains (i.e., the signal peptide-less part of the exported protein), that represent the final native states of these proteins, have evolved inherent properties that make them distinct from soluble cytoplasmic polypeptides. Collectively, the secretome is more flexible and disordered, folds more slowly, acquires a limited repertoire of very stable structures, comprising a few folds enriched in all β and avoiding the topologically more complex α/β folds. Ribosome-bound chaperones recognize, bind and actively sort a fraction of the exportome away from the cytoplasmome, and guide it to the membrane for export.

Taken together with experimental evidence (Chatzi et al., 2017; Sardis et al., 2017; Tsirigotaki et al., 2018), we propose that the secretome has developed slow folding and enhanced and extreme disorder. This reflects the collective evolutionary pressure of avoiding premature cytoplasmic folding, optimizing and securing TM crossing, being able to properly fold after secretion and responding to specific cell envelope functions. As not all protein structures are amenable to overcoming these demands, the structural landscape of the secretome is limited. These findings reveal a previously unsuspected evolutionary choice with wide implications. Secretome polypeptides rely primarily on their specific intrinsic features to delay their folding and promote disorder; extrinsic factors, such as chaperones, only modulate this repertoire.

## Materials and Methods

See Supplementary Materials and Methods for detailed information.

### Topological Annotation and Analysis of the *E. coli* K-12 Proteome

The STEPdb 2.0 database (updated from version 1.0; Orfanoudaki and Economou, 2014^1^), accessed through a mySQL management system, contains the *E. coli* K-12 “reference proteome” (MG1655/ATCC47076; UniProt Proteome ID UP000000625, 26/11/2017) with updated name and topological annotations (Supplementary Tables S1–S3), a new uniform naming scheme (see Supplementary Materials and Methods) and prediction tools and databases (Orfanoudaki and Economou, 2014; Orfanoudaki et al., 2017). All datasets are UniProt-referenced, in downloadable spreadsheets.

Using the CD-HIT algorithm (Li and Godzik, 2006) redundant protein sequences were removed at 90% sequence identity. The remaining 4247 proteins were then analyzed after removing signal peptides (510 proteins), unless specified otherwise. Nucleotide sequences corresponding to UniProt IDs were obtained from the European Nucleotide Archive.

mRNA abundance, translation efficiency and mRNA half-lives were obtained from ribosome-profiling (Li et al., 2014) and genome-wide transcriptomic microarray analyses (Esquerre et al., 2015). Average transcript decoding times were calculated based on decoding time scales (Dana and Tuller, 2014).

Relative frequency of amino acids (Polar: D, E, K, H, R, Q, N, S, C, T, Y, W; hydrophobic: I, L, V, F, Y, W, H, T, C, G, A, M and K; small: G, S, A and C; Taylor, 1986) and physicochemical properties of sequences were calculated with in-house scripts. pI was calculated using the IPC isoelectric point calculator (“EMBOSS” pKa set; Kozlowski, 2016).

Intrinsic disorder was predicted using IUPred2 (Meszaros et al., 2018) or the MobiDB aggregator (Piovesan et al., 2018). GRAVY (average hydropathy) was calculated based on the arithmetic average of the Kyte-Doolittle (K&D) score of each residue. Aggregation propensities and APRs and gatekeeper residues were predicted using TANGO (Fernandez-Escamilla et al., 2004). Predicted propensities for secondary structure acquisition such as α-helix, β-sheet, backbone dynamics and early folding predictions were derived as described previously (Cilia et al., 2013; Raimondi et al., 2017). Additionally structural secondary structure content was obtained from UniProt annotations based on a consensus between PDB structures. Other properties, such as proteome thermostability and structures, protein abundance and analysis and others, were obtained are described in Supplementary Materials and Methods. Protein structural classification was obtained from SUPERFAMILY (Gough et al., 2001). The machine learning tool JAD Bio (version 0.7; Borboudakis et al., 2017) was used for feature selection upon classification of K-12 proteins into cytoplasmic or secreted topology groups.

### Exploratory Analysis and Visualization

All parsing, mapping, data pre-processing and calculations were performed by scripts written in house in Python 2.7.10, unless otherwise mentioned. Statistical analysis was performed using R free statistical software version 3.3.1 (Supplementary Materials and Methods). Most of the graphical outputs were created using ggplot2 2.1.0.

## Results

### Updated Topological/Structural Annotation of the *E. coli* K-12 Proteome

We previously annotated the subcellular topologies of the K-12 proteome (Supplementary Table S1; STEPdb; Orfanoudaki and Economou, 2014). Several entries were updated due to additional experimental evidence, genome/proteome re-annotation and changed entry names (Supplementary Tables S2, S3). New and corrected topological and structural information has been incorporated (Supplementary Tables S1–S3) and includes peripheral IM or exportome proteins that are longitudinally positioned at specific plasma membrane regions along the cellular axis (Supplementary Figure S1 and Supplementary Table S4). In total, 2930 annotations of 1292 proteins have been updated (Supplementary Table S2; see below) and structural and functional information was added to all 4313 proteins (Supplementary Table S3). By comparison, the currently available subcellular localization data in UniProt cover 2070 proteins (∼48%) and complete or partial PDB structures for 1466 proteins (∼34%; see below).

### Topologically Correct Folding Requires Intrinsic and Extrinsic Factors

Proteins acquire folded states in one of the cell’s compartments (Figure 1A; De Geyter et al., 2016; Tsirigotaki et al., 2017) through optimal interactions of “intrinsic” (Figure 1B, red) and “extrinsic” (green) factors. Intrinsic factors are physicochemical properties of the polypeptide itself, while the extrinsic ones refer to the environmental factors. Intrinsic primary structural features define propensities for folding, solubility, aggregation, interactions and targeting. Thus, N-terminal or internal signals guide binding to DNA (405 proteins) or crossing (548) or embedding (970) in the IM (Figure 1A and Supplementary Table S1; De Geyter et al., 2016; Tsirigotaki et al., 2017).

Extrinsic factors bias equilibria: translation rates, abundance, metal ions and prosthetic groups, folding solvent and temperature (Figure 1B, green) and proteostatic machineries (Figure 1B, PM and Supplementary Tables S5, S6). Proteostatic machineries are being actively probed and rather well understood (De Geyter et al., 2016). In contrast, intrinsic features remain less clear and will be analyzed below.

### Amino Acid Content and Physicochemical Features

**FIGURE 2:**
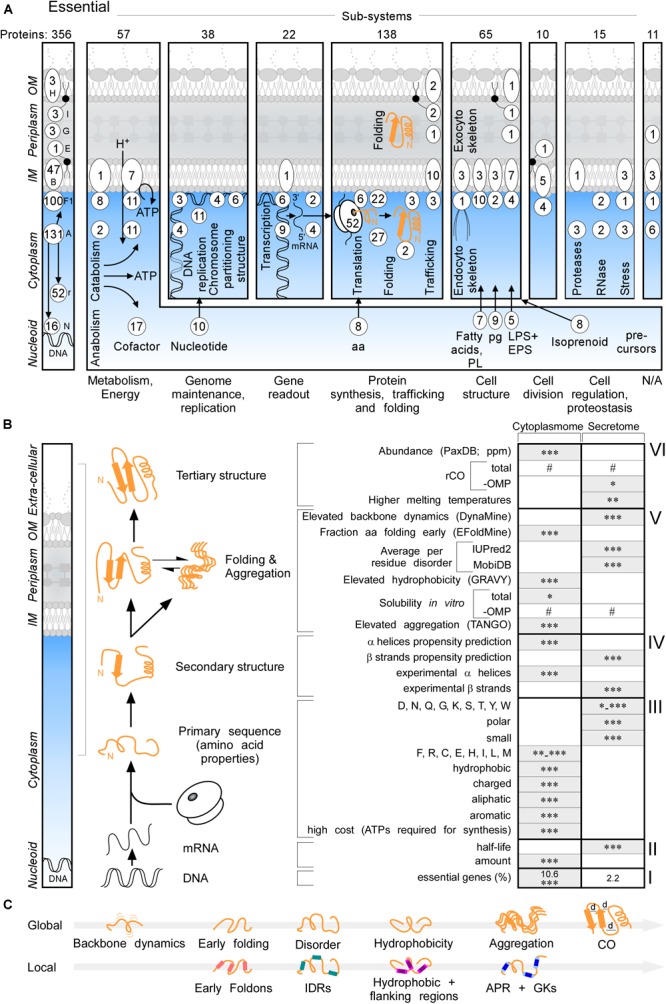
Essential proteins and global features of the K-12 proteome. **(A)** Three hundred and fifty-six essential proteins (Goodall et al., 2018) of different functional sub-systems (see bottom) distributed in different topology locations. #The sum of essential proteins in each panel. **(B)** Comparison of the indicated global intrinsic features of cytoplasmic versus secreted proteins. Schematic representation a cell locations (left) and different steps of protein synthesis and folding (middle). mRNA characteristics (adapted from Li et al., 2014; Esquerre et al., 2015); Solubility (adapted from Niwa et al., 2009). See text for details. Gray boxes: higher values in one group compared to the other. Statistical analysis was done using Kruskal–Wallis Test or Fisher’s exact test: ^∗^*p* < 0.05; ^∗∗^*p* < 0.01; ^∗∗∗^*p* < 0.001, #*p* ≥ 0.05. **(C)** Global (top) and Local (bottom) features described in this study in order of appearance. Local regions characterized are indicated in color: pink for early foldons, cyan for IDRs, magenta for hydrophobic regions and light magenta for the flanking regions, blue for APRs and light blue for gatekeepers. rCO, relative contact order; d, distance between amino acids; OMP, outer membrane proteins; IDR, intrinsically disordered region; APR, aggregation prone region; GK, gatekeepers; N/A, unknown.

Essential proteins comprise 10.6 and 2.2% of the cytoplasmome and exportome, respectively (Figure 2A, left and B,I). 57 of the 356 essential proteins of K-12 (Goodall et al., 2018), are exported. When considering functional cellular sub-systems (Figure 2A, right; containing both cytoplasmic and exported essential components, e.g., cytoplasmic enzymes that provide cell envelope precursors), 37% of all essential proteins have exportome and cell envelope related functions, and 60% of all essential cytoplasmic chemistries occur at the IM. This reflects the highly integrated and coordinated nature of cell structure, metabolic conversions and information flow.

Cytoplasmome mRNAs have higher concentrations but lower half-lives than those of the secretome (Figure 2B, II; Supplementary Figure S2A). mRNA concentration and half-life are negatively correlated (Nouaille et al., 2017). Cytoplasmic proteins have higher abundance (Figure 2B, VI; PaxDB; Wang et al., 2015) in keeping with, mRNA concentrations being positively correlated with protein abundance (Greenbaum et al., 2003).

To globally analyse primary sequences, we compared several primary and derivative physicochemical properties of the K-12 proteome (Figure 2B). The secretome is enriched in polar, small and disorder-promoting residues, the cytoplasmome on the other hand in hydrophobic, aliphatic, aromatic and charged residues (Figure 2B, III and Supplementary Figure S2D). Secreted proteins use significantly more residues that are energetically less costly to make (Figure 2B, III and Supplementary Figure S2D; Smith and Chapman, 2010). Secreted proteins (excluding OM proteins), are on average shorter than cytoplasmic ones (Supplementary Figure S2J). IM proteins and OM proteins are on average longer than cytoplasmic proteins (Supplementary Figure S2C). IM proteins are enriched in continuous stretches of hydrophobic residues, while OM proteins are not (Supplementary Figure S2D), since ∼8-residue β-strands with ∼4 non-continuous hydrophobic residues can cross the OM, forming β-barrels. IM proteins, but not the rest of the exportome, display higher pI (Supplementary Figure S2D), presumably due to charged residues providing TM topology cues (Schwartz et al., 2001).

The total secondary structure propensity (Cilia et al., 2013; Raimondi et al., 2017) and the actual content identified in solved structures (Figure 2B, IV; Supplementary Figures S2E,F), differ significantly between the two groups: cytoplasmome has more α-helix and secretome more β-stand content and are organized differently in folds (see below).

### Global Folding, Disorder, and Aggregation Characteristics

We next examined global chain flexibility and folding propensities in the proteome. First, we tested backbone dynamics using DynaMine (Cilia et al., 2013). Secreted protein backbones are significantly more flexible than cytoplasmic ones (Figure 2B, V); IM proteins are the most “rigid” (Supplementary Figure S3A).

Backbone dynamics agree well with average early folding propensities predicted using EFoldMine (Raimondi et al., 2017). Compared to the secretome, cytoplasmic proteins are predicted to fold earlier (as determined from the fraction of amino acids in each protein that is predicted to fold early; Figure 2B, VI and Supplementary Figure S3B), implying that the secretome is primed for slower folding.

Fast folders differ by various degrees of disorder from other polypeptides that partially or wholly lack folded structure, while remaining soluble (Dunker et al., 2001). Disordered proteins have been analyzed by experiments and predictions (van der Lee et al., 2014). Secreted proteins are predicted by IUPred2 (Walsh et al., 2015) or MobiDB (Piovesan et al., 2018), a database combining seven disorder predictors, to be more disordered than cytoplasmic ones (Figure 2B, V and Supplementary Figure S3C, lane 1–3 and lane 4–6, respectively; Tsirigotaki et al., 2018).

To gain insight into folding, and the aggregation side-reaction, of different topological groups, we also looked at K-12 proteome hydrophobicity using GRAVY (Kyte and Doolittle, 1982). Secretory proteins are less hydrophobic on average than cytoplasmic ones (Figure 2B, V and Supplementary Figure S2G). Solubility of 3153 K-12 proteins (including signal-peptide bearing secretory proteins), analyzed immediately after they had been synthesized in a cell free system, is distributed bimodally (low 20–30%; high 70–90% solubilities, Niwa et al., 2012). IM proteins are the most insoluble (96%; Supplementary Figure S2G; Niwa et al., 2009; Rawlings, 2016). Cytoplasmic proteins are more soluble (*p* < 0.05) than secreted ones, but this difference evens out when OM proteins are excluded (Figure 2B, V). Proteome aggregation is influenced by hydrophobic APRs and abundance/temperature, all promoting undesirable bimolecular collisions (see below). TANGO, a well-established tool for predicting the aggregation propensity of individual amino acids in the protein as well as identifying the APR and gatekeeper regions, predicts that secreted proteins are the least (Figure 2B, V) and IM proteins the most (Supplementary Figure S2G) aggregation prone.

Finally, we analyzed CO, a structural feature of folded proteins that has been correlated with fast (low CO) and slow (high CO) folding (Plaxco et al., 1998; Faisca et al., 2012; Baiesi et al., 2017) although this correlation is not fully understood and requires further study (Ivankov et al., 2009; Faisca et al., 2012; Baiesi et al., 2017). CO represents average distances in a aminoacid sequence between neighbors in the 3D structure (Figure 2B, VI). We calculated CO normalized by protein length, termed “relative CO” and hereafter rCO, for 1407 proteins with experimentally solved structures (Supplementary Tables S7, S8). The secretome (including OM proteins) and cytoplasmome have comparable rCO values but the secretome, excluding OM proteins, has higher rCO (*p* < 0.05), indicating that longer contacts are formed inside the soluble secreted proteins and might fold more slowly. IM proteins have the lowest rCO (Supplementary Figure S2H).

Secretory proteins are significantly more thermostable than cytoplasmic ones (Figure 2B, VI and Supplementary Figure S2H; Leuenberger et al., 2017; Mateus et al., 2018), suggesting that the former may compensate for slow folding and enhanced disorder.

**FIGURE 3:**
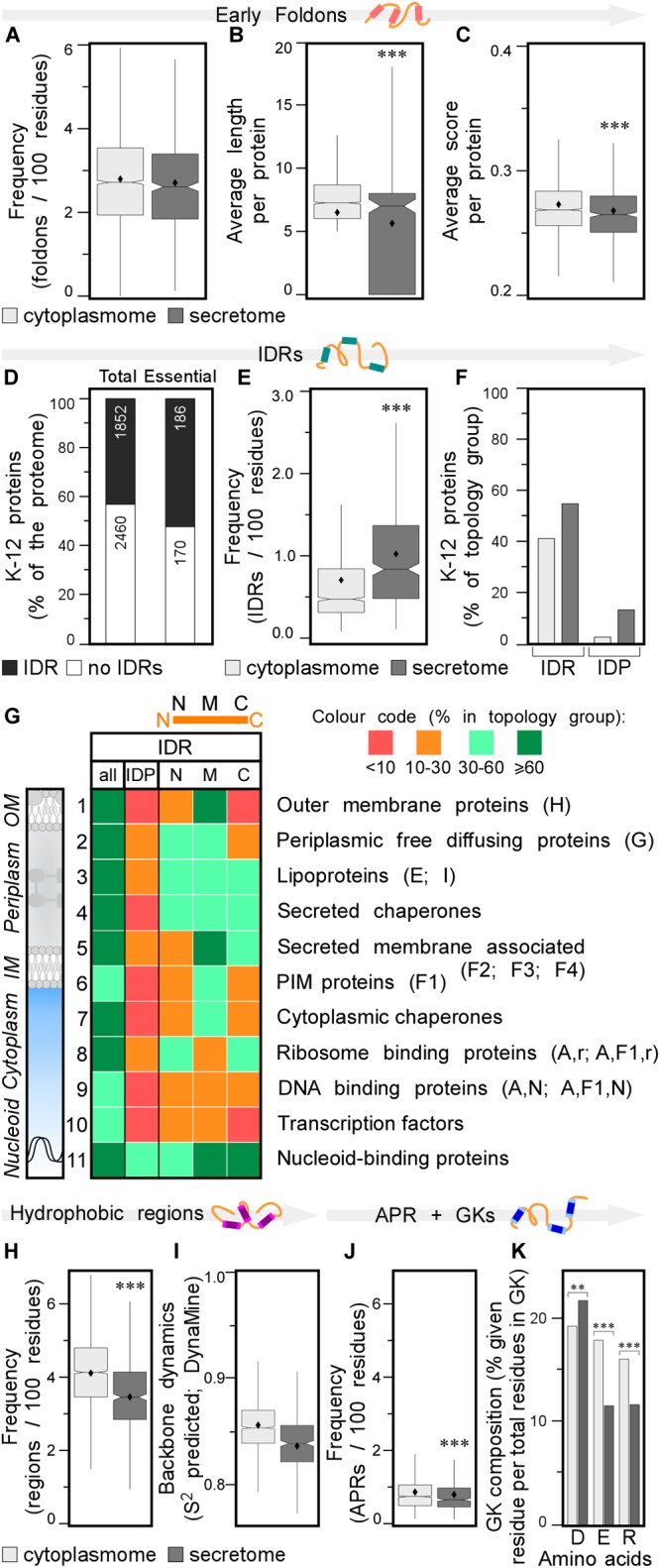
Local features of the K-12 proteome. **(A)** Frequency of early foldons in proteins (foldons per 100 amino acids). **(B)** Average length of early foldons per proteins. **(C)** Average score of early foldons per proteins. **(D)** Prevalence of proteins with IDRs in K-12 (numbers of proteins are indicated in the bar). **(E)** Frequency of IDRs in proteins (IDRs per 100 amino acids). **(F)** Percentage of proteins having IDRs (left) or being IDPs (right). **(G)** Heat-map of overall IDR occurrence and in N terminal (N), middle (M), C terminal (C) parts of the protein as well as the IDPs for different subcellular topology classes. Classes are noted on the right with STEPdb topology symbols in parenthesis. **(H)** Frequency of hydrophobic regions in proteins (regions per 100 amino acids). **(I)** Backbone dynamics prediction for hydrophobic regions (DynaMine; Cilia et al., 2013). **(J)** Frequency of APRs in proteins (APRs per 100 amino acids). **(K)** Relative frequency of amino acids in gatekeepers. Statistical analysis was done using Fisher’s exact test comparing cytoplasmic protein group to either IM or secreted protein groups: ^∗^*p* < 0.05; ^∗∗^*p* < 0.01; ^∗∗∗^*p* < 0.001. IDR, intrinsically disordered region; PIM, peripheral inner membrane; APR, aggregation prone region; GK, gatekeepers.

The above global features (Figure 2C, Global) were probed further at the level of local elements and their spatial distribution (Figure 2C, Local; Figure 3).

### Early Foldons and Intrinsically Disordered Regions

Early foldons are short stretches of amino acids predicted to provide backbones with a folding “roadmap” in foldon-dependent protein folding theories (Englander and Mayne, 2017). These regions would lead to fragments of lower free energy structures and eventually a stable fold downstream (Maity et al., 2005; Raimondi et al., 2017). Early foldons defined here to be at least five residues long, are found with similar frequencies in the cytoplasmome and secretome (Figure 3A; see Supplementary Materials and Methods and Supplementary Tables S9, S10). Yet, secretory protein early foldons are on average shorter and cytoplasmic ones have significantly higher EFoldMine prediction scores (Figure 3B,C and Supplementary Table S10).

Many proteins comprise short (5–19 amino acids) or long (≥20 amino acids) IDRs (Necci et al., 2016). In IDPs coverage by IDRs is extensive (IDPs are defined as proteins covered by IDRs for ≥30%; Figure 1B and Supplementary Figure S3D) and they have longer and more disordered IDRs (Supplementary Figures S3E,F, respectively). First, we tested how IUPred2 predicts disorder in an experimentally determined dataset, using the Disprot database (Piovesan et al., 2017), which lists 44 K-12 polypeptides with experimentally determined IDRs, including 22 IDPs (16 of them with IDRs of >50 amino acids; Tsirigotaki et al., 2018). We added to this list the experimentally defined, completely disordered YciG (Sardis et al., 2017), SodC and NrfB (Tsirigotaki et al., 2018) and the disordered CsgA curli amyloid fiber subunit (Evans et al., 2015). To cover the whole gamut of disorder (Dyson, 2011), we define here additionally: IFPs. These display flexibility that was experimentally determined by global HDX-MS (≥60% deuterium uptake; Tsirigotaki et al., 2018) or NMR (Prehna et al., 2012). IFPs include several exported proteins like the periplasmic glucose-binding protein (Tsirigotaki et al., 2018), the chaperone Spy (Quan et al., 2011; Tsirigotaki et al., 2018) and the extracellular YebF (Supplementary Table S10; Prehna et al., 2012). In general, IDPs are thought of as being more hydrophilic and having less structure in solution when compared to an average protein (Uemura et al., 2018). Many eukaryotic IDPs display a great variety of structural features including extended stretches of over-represented Gln or Ser residues (Dyson and Wright, 2005; Dunker et al., 2008; van der Lee et al., 2014; Uversky, 2016, 2019). In bacteria, only 35% of the experimental IDPs of K-12 show extreme hydrophilicity (Supplementary Table S9) and it was possible to crystallize 23 out of 26 of them yielding high coverage crystal structures.

To decide on a disorder predictor for proteome-wide analysis we tested several tools and run them against the experimentally determined disordered proteins from Disprot (Piovesan et al., 2017). We focused in particular on MobiDB (Piovesan et al., 2018) and IUPred2 (Meszaros et al., 2018) (see Supplementary Materials and Methods and Supplementary Table S9, columns S–U). However, the MobiDB consensus score is not numerical (only parametric, i.e., ordered or disordered). IUPred2, is highly specific at the expense of sensitivity (Meszaros et al., 2018; Necci et al., 2018), and returned results that correlated well with those obtained from MobiDB. In addition, IUPred2 can process large whole-proteome datasets and was used hereafter. To set performance expectations for IUPred2, we predicted disorder in the 54 experimentally characterized IDPs, IFPs and IDR-carrying proteins (Supplementary Table S9). Of these, 18 are bioinformatically defined as *bona fide* IDPs (i.e., ≥30% sequence coverage by IDRs of ≥5 residues), another 23 had ≥1 IDR and 13 had no predicted IDRs. IUPred2 does not predict six experimentally determined IDPs (23% false negatives). Clearly, both disorder predictions and experimental validations must be consulted.

Having evaluated the performance of IUPred2, we used it to re-examine proteome-wide disorder in K-12 (Supplementary Tables S9, S11; Dosztanyi, 2018). Forty-three percentage of the proteome and 52% of the essential proteins (Paliy et al., 2008), contain at least one IDR (see Supplementary Materials and Methods; Figure 3D, lane 1 and 2, respectively). Twelve percentage of the K-12 proteome has long IDRs (≥20 amino acids) and 4.4% of the proteome proteins are IDPs (189; including the experimentally determined disordered/flexible proteins that IUPred2 did not predict; Supplementary Table S9). The secretome has more IDRs than does the cytoplasmome (1.1 vs. 0.7/100 residues; Figure 3E). 41.3% of the cytoplasmome and 55.5% of the secretome, respectively, have ≥1 IDR (Figure 3F, left and Supplementary Figure S3F).

The difference between cytoplasmome and secretome is striking when comparing their IDPs: 3.0 and 13.7% of each group are predicted IDPs, respectively (Figure 3F, right; Supplementary Table S9; Roderer and Glockshuber, 2017; Tsirigotaki et al., 2018). Secreted IDPs are involved in transport-, division-, motility-related processes and cellular responses to stress and often function as enzymes. Disorder might be important in protein-protein or protein-substrate interactions (van der Lee et al., 2014). IM proteins are the least disordered (Supplementary Figures S2G, S3G): only 1.9% are IDPs and an additional 24% have ≥1 IDR (Supplementary Figure S3G). TMs in IM proteins are so ordered that TM, hydrophobicity and IUPred2 disorder predictions complement each other (see below; Supplementary Figure S4).

IDR-containing proteins were next analyzed separately from IDPs. The differences in disorder between the two groups persist even if all IDPs are removed from the dataset (data not shown), indicating that disorder is inherent and widespread in the secretome (Tsirigotaki et al., 2018). This explains why it was such a strong enough predictor of secretome polypeptides (Orfanoudaki et al., 2017).

Finally, we classified IDRs with respect to their location within a protein sequence as: N-terminal (starting in the first 30 residues), middle or C-terminal (extending till the last 30 residues) (Figure 3G, top). Certain subcellular topology groups are enriched in specifically positioned IDRs (Figure 3G). For example, over 2/3 of OM proteins (row 1) have, mostly middle IDRs. Freely diffusing periplasmic proteins often have N-terminal and middle IDRs (row 2). Lipoproteins (row 3) are the most disordered subcellular group in K-12: 19% are IDPs; 52% have N-terminal IDRs (row 3; Zuckert, 2014; Asmar et al., 2017). Over 2/3 of all secreted peripherally membrane-associated proteins (i.e., Figure 1A, classes F2–4) and almost half of the peripheral IM proteins (Figure 1A, F1) have mid-region IDRs (Figure 3G, row 5 and 6, respectively). Ribosomal (Peng et al., 2014) and ribosome-associated proteins are disorder-enriched (row 8); 1/3 of them have N- and C-terminal IDRs. DNA-associated proteins (row 9) often have middle IDRs. Transcription factors (row 10) and nucleoid-binding proteins (row 11) from this group are especially enriched in disorder, as previously observed for eukaryotic DNA-binding proteins (Lobley et al., 2007). Chaperones carry many IDRs (rows 4, 7; van der Lee et al., 2014): 2/3 have predicted, usually short middle or C-terminal IDRs (Supplementary Table S9). Periplasmic chaperones (row 4) are even more disordered than cytoplasmic ones (row 7).

### Hydrophobicity, Solubility, and Aggregation

Protein folding in aqueous environments exploits equilibria between solubility (common in final folded states) and aggregation (often manifested when folding intermediates expose APRs (Beerten et al., 2012).

Secreted proteins have fewer continuous hydrophobic patches per 100 residues (Supplementary Table S12; Tsirigotaki et al., 2018), that are shorter and less hydrophobic, than those of cytoplasmic proteins (Figure 3H and Supplementary Figures S3H,I) and are similarly distributed in the two groups (Supplementary Figure S3J). Hydrophobic patches in secretome polypeptides (Figure 3I) and their flanking quintapeptides (Supplementary Figure S3K), have higher backbone dynamics than the corresponding elements in the cytoplasmome. As expected, IM proteins have the most hydrophobic patches, some functioning as TMs. N-terminal hydrophobic patches are particularly common in IM proteins (Supplementary Figure S3J), presumably contributing to their recognition by SRP (see below).

A subset (28%) of the hydrophobic patches (of ≥5 amino acids) are APRs and satisfy β-strand formation (2–4/globular protein; Supplementary Table S13; Fernandez-Escamilla et al., 2004). APR length, prediction scores and distribution in the primary sequence are similar for secreted and cytoplasmic proteins (Supplementary Figures S3L–N), but cytoplasmic proteins are more aggregation prone and have significantly more APRs, than do secreted ones (0.86 vs. 0.79/100 amino acids, respectively; Figure 3J). IM proteins have the most, frequently N-terminal, APRs (Supplementary Figure S3N) compared to the cytoplasmome and secretome.

Gatekeeper residues flank and reduce the aggregation of APRs (Beerten et al., 2012). Gatekeepers differ for the cytoplasmome (more Glu and Arg), secretome (more Asp) and IM proteins (more Pro/Gly and less Lys; Figure 3K and Supplementary Figure S3O and Supplementary Table S13; Beerten et al., 2012). Collectively, differences in residues that flank hydrophobic patches may contribute in enhancing solubility of the secretome in non-folded, presecretory states (Tsirigotaki et al., 2018).

### The K-12 Foldome

**FIGURE 4:**
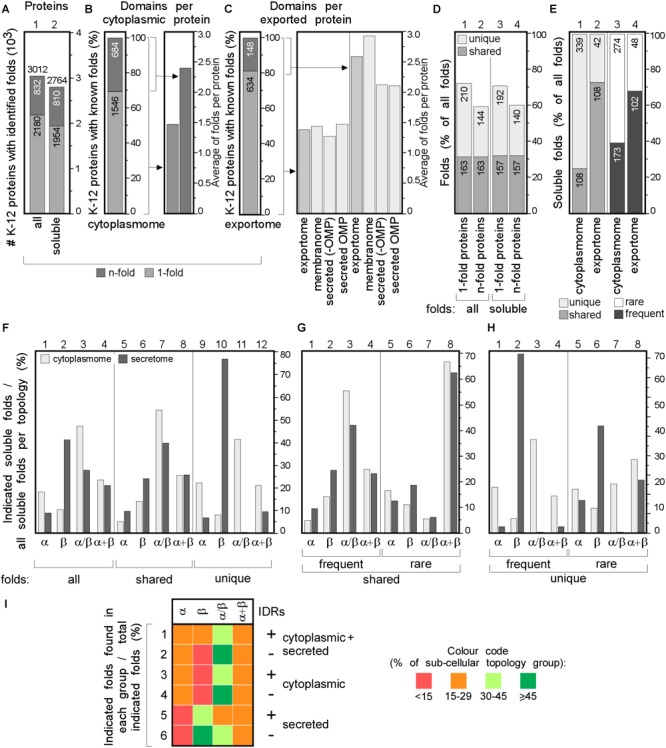
Structural differences in cytoplasmic-secreted proteomes. **(A–C)** Structural fold analysis (numbers of proteins are indicated in the bars). **(A)** K-12 proteome with al identified folds or only soluble folds for one (onefold) or multiple (n-fold) proteins. **(B)** Domains per cytoplasmic protein. Left: percentage of proteins that carries only one or more folds. Right: average folds per protein for all cytoplasmic proteins or n-fold proteins. **(C)** Domains per exported protein. Left: percentage of proteins that carries only one or more folds. Right: average folds per protein for exported proteins. **(D)** Percentage of folds unique to either cytoplasmic or exported proteins or shared among the two groups. **(E)** Soluble folds in cytoplasmic or exported proteins based on carrying one or more folds (left) and carrying rare (≤3 times in the proteome) or frequent (≥4 times in the whole proteome) folds (right). **(F)** Indicated soluble fold classes in cytoplasmic and secreted proteins. On the Left: all soluble folds in each topology group; middle: unique and right: shared folds between the two topology groups. Unique **(G)** and shared folds **(H)** are divided in rare and frequent folds and plotted for cytoplasmome and secretome in α, β, α/β and α+β classes. **(I)** Heat-map of fold class occurrence in the proteins with (+) and without (–) IDRs for different topology classes. OMP, outer membrane proteins; IDR, intrinsically disordered region; N/A, none found.

We next classified and compared fold families in the topology groups using (SCOPe and SUPERFAMILY; Figure 4 and Supplementary Tables S3, S14; De Geyter et al., 2016). Proteins were classified into classes (corresponding to secondary structure content), folds (groups of structurally similar arrangements of secondary structure, not necessarily evolutionarily related; e.g., DNA/RNA-binding 3-helical bundle) and super-families (groups of proteins with evolutionary relatedness; e.g., homeodomain-like) identified using Hidden Markov-Models (Wilson et al., 2007; De Geyter et al., 2016).

We focused on the following classes: α, β, α/β (mostly parallel β-sheets alternating with stabilizing α-helices.), α + β (α and β domains in tandem). Class f (membrane and cell surface proteins) was only used to extract soluble, non-membrane embedded folds. In total, we identified 517 unique folds in 3012 K-12 proteins (Figure 4A, lane 1). Four hundred and eighty-nine of these are soluble in 2764 proteins (Figure 4A, lane 2). Most folds are found in the cytoplasmome, the largest topological class. Thirty-one of the cytoplasmome and 19% of the secretome has proteins with more than one domain (n-folds; Figure 4B,C, left). Of these, 32% are shared between proteins with one or multiple domains. More IM proteins have multiple domains compared to cytoplasmic proteins (Figure 4B,C, right; Orfanoudaki et al., 2017).

The cytoplasmome and secretome share 157 soluble domain folds that are structurally distinct (Figure 4D). The exportome is structurally poorer: (a) it has fewer unique folds (42 vs. 339; Figure 4E, lane 1 and 2). (b) It has many frequent folds (≥4 times in the whole proteome; 68% vs. 39%; Figure 4E, lane 3–4). (c) Only 32% of its domains are rare (found 1–3 times in the whole proteome), compared to 61% of all cytoplasmome folds (Figure 4E, lane 3–4).

Cytoplasmic proteins are enriched in α (Figure 4F, lane 1) and α/β (lane 3) classes and the secretome in β (lane 2). This distribution is maintained in shared folds (with α being slightly more favored in the secretome; lane 5) but becomes highly exaggerated in unique folds: these are predominantly α, α/β and α+β in the cytoplasmome (Figure 4F, lane 11 and 12) but 80% are β (lane 10) and none is α/β (lane 11) in the secretome. Both frequent (Figure 4G,H, lanes 1–4) and rare folds (lanes 5–8), are responsible for the cytoplasmome/secretome differences, especially for β and α/β folds. Variations are detectable in both frequent and rare unique folds (Figure 4H) with the secretome being overwhelmingly enriched in β (lanes 2 and 6) and depleted of α/β (lane 3 and 7). Collectively, these data suggest selective pressure may account for the enrichment of specific folds in specific subcellular compartments. For this comparison we excluded the IM proteins since they can contain soluble folds that face the cytoplasm and therefore, would not have been subjected to the same evolutionary pressure as the soluble domains of the secretome. The differences between the cytoplasmome and the exportome for different groups of folds are less prominent (Supplementary Figure S5).

The relation of disorder propensities, backbone dynamics and structural preferences of the cytoplasmome and the secretome was also examined. Enrichment of α folds and of α + β in the cytoplasmome, is independent of disorder status. All proteins with IDRs are enriched in α/β folds (Figure 4I, rows 1–6 and Supplementary Table S9). β folds are less common in IDR-containing cytoplasmic proteins than in ones without IDRs (12% vs. 9%; rows 1 and 2), while secreted proteins without IDRs have more β folds (rows 5–6). This implies that in certain cases fold distribution correlates with disorder propensities.

**FIGURE 5:**
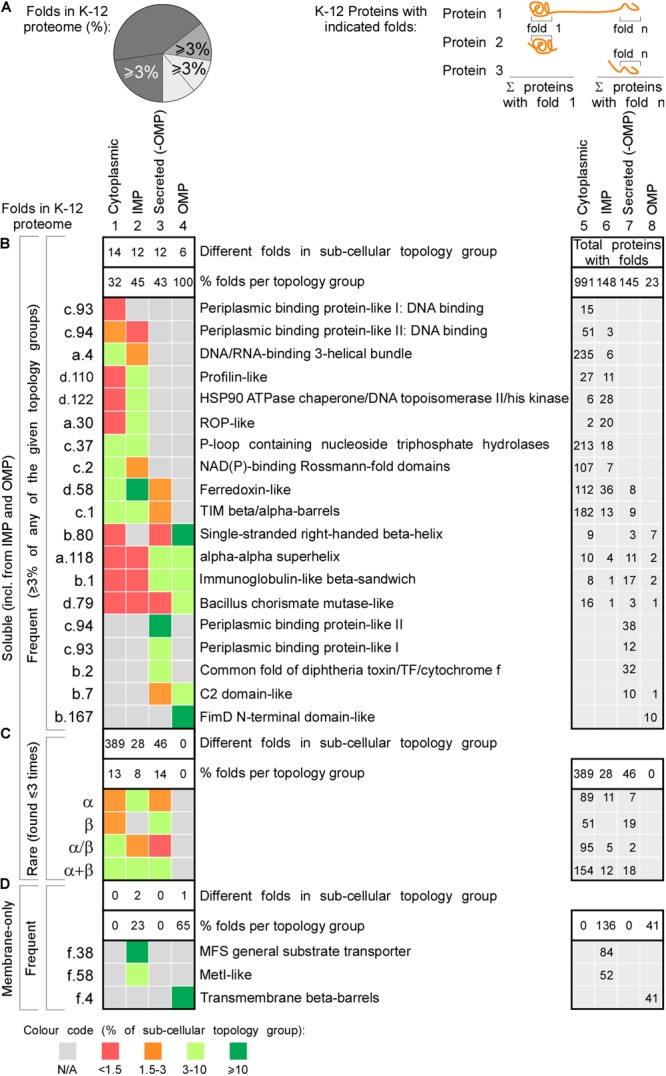
Functional classes of the most frequent protein folds of K-12. Heat-map of occurrence of the most frequent and rare folds in different topology classes. **(A)** Left: cartoon representation of percentages depicted in the heat map (**B–D** on the left). Percentage of folds that represent ≥3% of all the folds found in each topology class (cytoplasmome, IMP, secretome without OMPs or OMPs). Such folds are included in panels **(B,D)**. Right: cartoon representation of the numbers depicted in the table on the right. Each number is how many proteins were found having the respective fold. Empty cells when no proteins were found with that fold. **(B)** Nineteen soluble folds are shown that each represent ≥3% of folds found in a specific topology group. Left: fold abbreviation; right: full name. On the very right the sum of proteins having each fold is indicated. The absolute number of folds found in each subcellular topology group is noted in the upper white box and its corresponding percentage below it in the bottom white box. The upper white box on the right depicts the total protein sum in each topology. **(C)** The sum of four classes of folds that are rare and their distribution in the topology groups. **(D)** The membrane folds that represent minimum 3% of folds found in different topology groups. IMP, inner membrane proteins; OMP, outer membrane proteins; N/A, none found.

We next analyzed the most popular K-12 folds, i.e., those representing ≥3% of the folds in either cytoplasmome, IM proteins, secretome (excluding OM proteins) and in OM proteins (Figure 5A and Supplementary Table S14). In total, only 17 frequent folds comprise 34.8% of the K-12 foldome (Figure 5B). Only five different folds contribute ∼30% of the cytoplasmic foldome (Figure 5B, column 1, light green) and >50% of them bind nucleotides (DNA binding, ATPase, Rossmann-fold). These are completely absent from proteins of the nucleotide-free periplasm. Just six and five different folds, respectively, comprise almost half of all soluble IM proteins and secretome folds (Figure 5B, column 2 and 3 in left table, green). 8–14% of each topology group represents the rare folds (found 1 to 3 times in each subcellular topology group; Figure 5C). The most frequent IM protein folds are the MFS general substrate transporter and the MetI-like fold of ABC transporters (in 84 and 52 proteins, respectively; Figure 5D). >60% of OM proteins contain only six soluble folds. TM β-barrels are the only membrane-spanning fold present in OM proteins (Figure 5D).

N- and C-termini may become proximal in the final 3D folded states but no significant distance differences were detected between topology groups (Supplementary Figure S6 and Supplementary Table S3).

### Translation Rate and Protein Abundance

Translation rates and folding speed finely tune cellular folding versus aggregation (Buhr et al., 2016). Translation decoding time and, therefore, translation efficiency (i.e., translation rate normalized per protein abundance, see section “Materials and Methods”), is comparable between secretome and cytoplasmome-encoding mRNAs (Supplementary Figure S2B; Dana and Tuller, 2014), while IM proteins, have much lower translation efficiency and higher decoding times (Supplementary Figure S2B). This has been also associated with low solubility (Niwa et al., 2009) and may offer more SRP interaction opportunities (see below).

**FIGURE 6:**
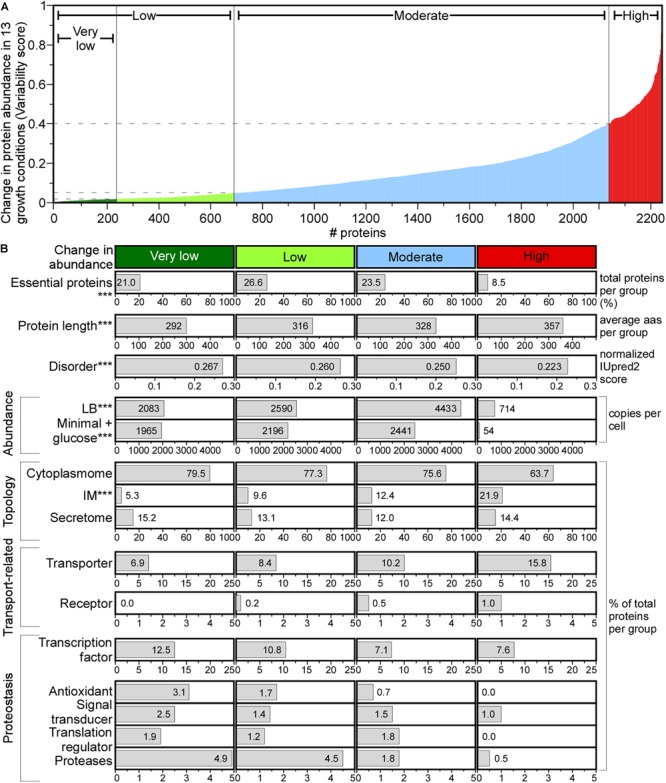
Change in protein abundance across different media. **(A)** Change in protein abundance plotted using variability score (see section “Materials and Methods”) for each protein. Proteins were classified in the indicated groups. **(B)** Protein features quantified for each group. Statistical analysis was done using Kruskal–Wallis Test or Fisher’s exact test: ^∗^*p* < 0.05; ^∗∗^*p* < 0.01; ^∗∗∗^*p* < 0.001 for the comparison between very low and high score groups. aas, amino acids.

Protein abundance influences aggregation and may lead to co-evolved properties and has been quantified in *E. coli* under multiple growth regimes (Lill et al., 1988; Matsuyama et al., 1992; Taniguchi et al., 2010; Soufi et al., 2015; Schmidt et al., 2016; Caglar et al., 2017). The 2354 proteins (57% of the total proteome) of K-12 strain BW25113, a close relative of MG1655, were quantified (Grenier et al., 2014). Here, we extrapolated the abundance of 2353 homolog proteins in MG1655, in 13 conditions that did not involve stress or protracted growth (Figure 6A and Supplementary Table S15). Cytoplasmic and secreted protein concentrations are similar, while IM proteins are found at much lower levels (Supplementary Table S3; Wang et al., 2015; Schmidt et al., 2016).

A variability score (VS, see Supplementary Materials and Methods) was used to define four classes of protein abundance change: very low, low, moderate and high (Figure 6A). The abundance of 10.4% of the proteome remains constant (Figure 6A, very low), and of 8.6% changes substantially. The variable abundance proteins are less commonly essential, include many IM proteins, are less efficiently translated and are the least abundant (Figure 6B; Serohijos et al., 2012). Many transporters and metabolic enzymes of highly variable abundance, are activated in LB more than in the minimal medium supplied with a single carbon source. Proteins that undergo little abundance changes among different growth conditions are shorter, have more acidic and charged amino acids, lower pI and higher disorder and include many cytoplasmic proteins (79.5%) compared to the highly variable abundance group, while secretome proteins are distributed equally in all groups (Figure 6B).

Functionally, house-keeping proteins (e.g., transcription and antioxidant factors, proteases, signal transduction and translation regulation functions) are of constant to moderately changed abundance (e.g., Sec pathway subunits; Supplementary Table S15) and some are completely absent from the variable abundance group (Figure 6B). Transporters and receptors are enriched in the variable group (Ashburner et al., 2000).

### Chaperone Mediated Sorting *in statu nascenti*

**FIGURE 7:**
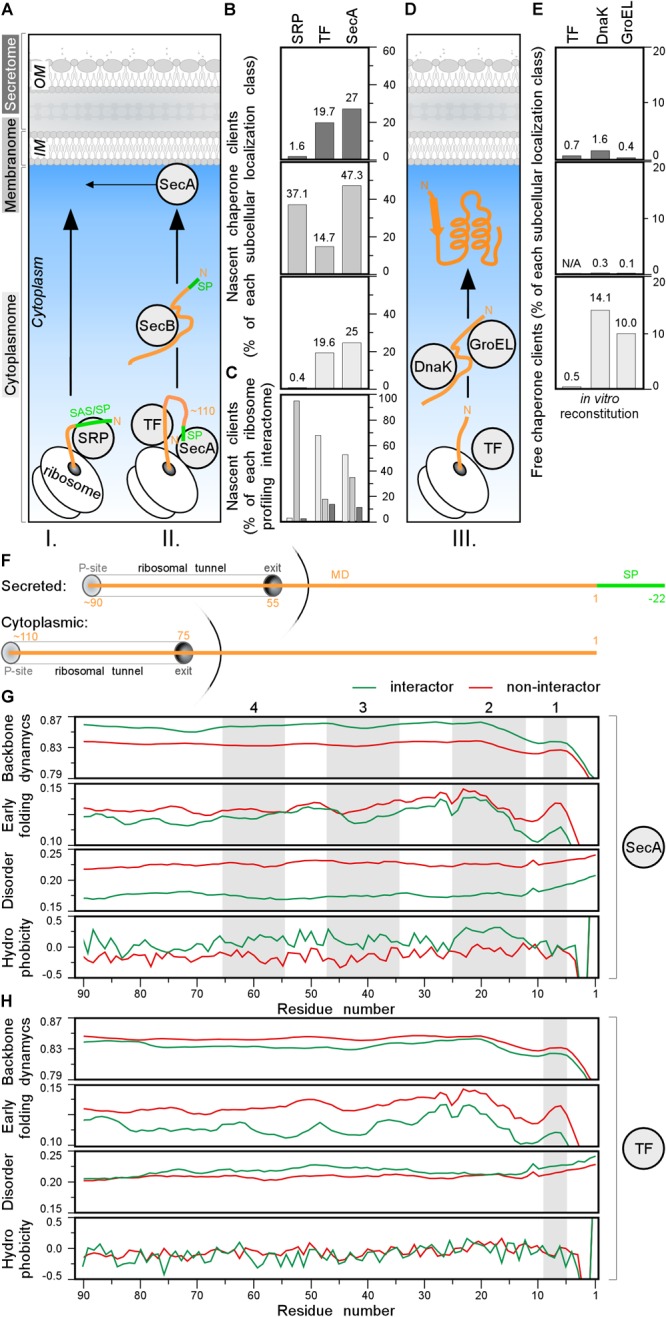
Chaperone clients and their features. **(A)** Left: representation of different topology groups in gray scale that is used in the graphs. Right: cartoon representation of different chaperones interacting with nascent chains co-translationally. **(B,C)** Nascent interactomes of the indicated chaperones in each subcellular localization group (Supplementary Table S16). Nascent interactomes determined by ribosome profiling (Oh et al., 2011; Schibich et al., 2016; Huber et al., 2017) were re-analyzed (see Supplementary Materials and Methods). **(B)** Nascent interactors of each subcellular localization class are plotted relatively to the total population of each class. **(C)** Relative distribution of the indicated nascent interactomes in the three subcellular localization classes [color coding as in panel **(A)**]. **(D)** Cartoon representation of different chaperones interacting with nascent chains post-translationally. **(E)** Interactomes of the indicated chaperones in each subcellular localization class, were identified as described (Niwa et al., 2012; Supplementary Table S17). **(F)** Cartoon representing where the chain is when 110 residues are synthetized for secreted (top) and cytoplasmic (bottom) proteins. P-site is peptidyl-site, followed by ribosomal tunnel and its exit. The corresponding length of the nascent chain is indicated in orange. **(G,H)** Average score of N-terminal residues (indicated on the *x*-axis) of backbone dynamics (DynaMine), early folding (EFoldMine), disorder (IUPred2) and hydrophobicity (Kyte-Doolittle scale) for SecA **(G)** or TF **(H)** interactors (green) and non-interactors. MD, mature domain; SP, signal peptide.

Ribosome-bound chaperones, interact with nascent chains as soon as the latter emerge from ribosomes and bias nascent polypeptide destinations (Figure 7A, step I. and II.; Solbiati et al., 1999; Bienvenut et al., 2015). The ribonucleoprotein SRP recognizes N-terminal hydrophobic TMs of IM proteins and a few, hydrophobic, secretory signal peptides (Figure 7A, step I; Tsirigotaki et al., 2017) and binds its FtsY receptor, to associate to membrane-embedded SecYEG channels co-translationally (Saraogi et al., 2014). SRP binds to empty ribosomes with a *K_d_* of 70 nM, that becomes higher (0.7–1.5 nM; IM proteins) or lower (200–800 nM; secretory proteins) when nascent proteins emerge (Bornemann et al., 2008, 2014). TF (Maier et al., 2003) and SecA (Huber et al., 2011) bind to empty ribosomes with a *K_d_* of ∼1 μM. SecA, may also associate with nascent secretory polypeptides (*K_d_* < 0.5 μM) and guide them for secretion possibly even co-translationally, after ∼100–110 residues are synthesized (Oh et al., 2011; Huber et al., 2017). Given their cellular concentrations (40 μM TF and ∼5 μM SecA; Tsirigotaki et al., 2017) and their shared ribosome docking sites (Huber et al., 2011), nascent chains are more likely to encounter TF as they emerge.

Co-translational nascent chain interactions are commonly determined *in vivo* using selective ribosome profiling (Oh et al., 2011; Becker et al., 2013; Schibich et al., 2016). mRNA sequences are compared between the cellular translatome and specific ribosome-bound factor translatomes, isolated by (immuno)affinity purification. Such studies revealed competition between the three ribosome-bound chaperones and a complex landscape of a few overlapping and several non-overlapping clients (Supplementary Table S16; Bornemann et al., 2014; Ariosa et al., 2015). These interactions generate complex equilibria *in vivo* that are regulated additionally by partner concentrations (Supplementary Table S5) and translation speed (Supplementary Table S3).

Three hundred and seventy-nine co-translational SRP interactors were defined (see Supplementary Materials and Methods; Schibich et al., 2016), including 10 cytoplasmic, 360 IM proteins, and 9 secreted proteins (Figure 7B and Supplementary Table S16). Of 799 nascent TF interactors (Supplementary Table S16), 548 (>65% of TF interactome; 19.6% of total cytoplasmome) were cytoplasmic, 143 IM protein (67 of them shared with SRP; Supplementary Figure S7B) and 108 secretory (19.7% of total secretome; Figure 7B, top) of which only 21 OM proteins.

A similar approach identified 1305 nascent SecA interactors but only after they were additionally cross-linked (Figure 7B and Supplementary Table S16; Huber et al., 2017). Only 11.3% of these were secretory proteins, 35.2% were IM proteins and over half were with cytoplasmic and peripheral IM proteins (Figure 7B,C). Secretory SecA interactors included a third of its *bona fide* Sec clients but also seven flagellar and TAT secretory proteins that do not use the Sec system (Supplementary Table S16). This unexpected promiscuity toward cytoplasmic proteins and unrelated secretors may represent experimental false positives, or true, but weak, SecA interactions or meaningful interactions with unknown roles. SecA shares 145 interactors with SRP, 211 with TF and 41 with both (Supplementary Figure S7B).

To better define potential SecA and TF recognition features in nascent interactors, we focused on their N-terminal residues and compared them to those of other proteins. We plotted the average scores of backbone dynamics (DynaMine), early folding (EFoldMine), disorder (IUPred2) and hydrophobicity (Kyte-Doolittle scale) for every residue (starting at the first residue of the cytoplasmic protein or the first residue of the mature domain of secretory proteins until their approximately last, ribosome exposed 90th residue; Figure 7F). Throughout these ribosome-exposed N-terminal regions, SecA interactors display statistically significant differences from the non-interactors: less backbone dynamics, more slow folding residues, less disorder and islands with elevated hydrophobicity/folding (Figure 7G, gray islands 1–3 for both topological groups and four only relevant for cytoplasmome; Supplementary Figures S7G–J, left).

TF interactors, on the other hand, have more backbone dynamics, slightly more disorder islands and no obvious hydrophobicity islands compared to non-interactors (Figure 7H and Supplementary Figure S7G–J, right). TF interactors also fold later (EFoldMine) compared to non-interactors (Figure 7H). These differences between the SecA and TF nascent interactors might explain how these proteins are distinguished from the other nascent chains.

Collectively, SRP specifically recognizes many IM proteins co-translationally (Figure 7B,C and Supplementary Table S16) and may compete with TF and SecA for clients. Among the 505 secretory proteins, only ∼2, ∼20, and ∼30% appear to be SRP, TF and SecA nascent substrates, respectively (Figure 7B).

### Post-translational Cytoplasmic Chaperone Interactors

TF association might bias secretory protein choice for post-translational secretion and relay them to downstream chaperones, like SecB and SecA. SecB facilitates export of 13 of the 505 Sec-dependent secretory proteins (Figure 7A), and acts downstream of SecA/TF (Baars et al., 2006). Once exportome proteins have been selected out, cytoplasmic nascent chains complete translation and initiate folding (Figure 7D, III) alone or using foldases (e.g., GroEL, DnaK; Anfinsen et al., 1961; Anfinsen, 1973; Deuerling et al., 1999; Kim et al., 2013; Saio et al., 2014; Santra et al., 2017). Experimentally determined TF, DnaK, and GroEL “client” specificities yielded convoluted results (Supplementary Materials and Methods and Supplementary Table S17).

Here, we address only if potential cytoplasmic chaperone interact with the secretome during its cytoplasmic transit (Bochkareva et al., 1998) and only consider results from addition of TF, DnaK, and GroEL during cell free synthesis (Niwa et al., 2012). 1018 K-12 proteins refolded and/or became soluble without, and 521 with, chaperone help (Figure 7E and Supplementary Figure S7D and Supplementary Table S17; Niwa et al., 2009). More than a third of the latter interacted with 2 or 3 chaperones (Supplementary Figure S7C).

Most chaperone-solubilized proteins were cytoplasmic, while exportome solubilization was negligible (Figure 7E; middle and upper). This suggests that while foldases may act as “holdases” to prevent aggregation (Hoffmann et al., 2010), their influence on secretome polypeptide sorting and solubilization during their cytoplasmic transit to the translocase, is marginal. The inherent propensity of secretome polypeptides to retain non-folded/disordered states (Chatzi et al., 2017; Sardis et al., 2017; Tsirigotaki et al., 2018), predominates.

### Multiple Structural Features Differentiate the Subcellular Topology Groups

To objectively define the minimal-size set of contributing factors that differentiate between cytoplasmome and secretome, we used the machine learning tool JAD Bio (Borboudakis et al., 2017). We previously trained JAD Bio to predict differences between the cytoplasmome and the signal peptide-less mature domains of the secretome on the basis of N-terminal sequences (Orfanoudaki et al., 2017). JAD Bio employs an automated machine learning pipeline to produce a classification model from a given training dataset, and an estimate of its predictive performance (mean and confidence interval). At the same time, it performs multiple feature selection, i.e., identifies as many as possible minimally sized feature sets that collectively (multi-variately) contain all the information sufficient to produce an optimally predictive classification model.

First, we compared how different combinations of features perform in JAD Bio (Table 1). A comprehensive list of the 79 features (i.e., all the different protein properties; Supplementary Tables S3, S9), 8 of which dealt with disorder, resulted in 95.5% success, as measured by the Area Under the Receiver Operating Characteristic Curve (AUC), in distinguishing cytoplasmome from secretome polypeptides. To achieve this, JAD Bio selected 24 features (Supplementary Table S18). The most prominent amongst them (with the highest weight factor), were: disorder predictions, amino acid frequencies (e.g., methionine, glutamate, arginine are significantly more common in cytoplasmic than in secreted proteins), early foldon average score and hydrophobic regions (determined by EFoldMine and GRAVY tools, respectively) and the presence of certain folds.

**Table 1:** Performance of structural features to differentiate cytoplasmome from secretome using machine learning.

Features used	Average AUC ROC Curve	Accuracy	Precision for class “Cytoplasmic”	Precision for class “Secreted”	Reference
71 parameters + 8 disorder parameters	0.955 (0.942, 0.968)	0.931 (0.920, 0.942)	0.910 (0.885, 0.936)	0.794 (0.745, 0.840)	This study, Supplementary Table S18
71 parameters	0.955 (0.941, 0.967)	0.938 (0.925, 0.949)	0.913 (0.889, 0.941)	0.848 (0.801, 0.891)	This study
8 disorder parameters	0.783 (0.754, 0.811)	0.796 (0.777, 0.814)	0.837 (0.821, 0.853)	0.414 (0.367, 0.457)	This study

*Protein structural features in different combinations and their performance in contributing to differentiating between subcellular topology groups using the JAD Bio machine learning tool (Borboudakis et al., 2017) and Supplementary Table S18. Accuracy describes all correctly classified cases, while precision describes only the true positives among all the positive cases. Seventy-one parameters define the features as determined in Supplementary Tables S3, S9, excluding the eight disorder related features, which are treated as a separate class. Disorder parameters are IUpred2 score (normalized by length), IDRs per 100 amino acids, their coverage of the protein length (in %), their average length and score per protein and disorder group classification (IDP or IDR or no IDRs predicted). Shown are average of each score, followed by 2.5% low confidence and 97.5% upper confidence shown in brackets. aas, amino acids; AUC ROC, area under the receiver operating characteristic curve.*

Since the disorder prediction score was the most significant feature that JAD Bio used for classification, we extracted all of the disorder-related features (e.g., IDR frequency, average length, average scores per protein) and run them alone in the JAD Bio analysis (Table 1, “8 disorder parameters”). This resulted in a worse precision of secretory protein classification, indicating that disorder is combined with more features to successfully predict if a proteins belongs to the cytoplasmome or secretome. Attesting to this, classification accuracy and precision were improved when disorder parameters were excluded. In this case, coverage by hydrophobic regions was selected as an extra variable.

Our current dataset that includes more extensive structural features, separates the cytoplasmome from the secretome better than the best classification model (#M22) of the MatureP classifier that we previously developed (91.5% success; Orfanoudaki et al., 2017) and that was also using disorder and amino acid compositions.

We concluded that the structural features selected by the machine learning tool are prominent descriptors of the structural differences between the two cytoplasmome and secretome groups.

## Discussion

Understanding cellular systems requires comprehension of how their proteomes are compartmentalized. Moving physico-chemically heterogeneous aminoacyl polymers into and across biological membranes requires four main logistics solutions to achieve “secretability”: (a) management of chain “flexibility,” to prevent premature cytoplasmic folding for the exportome but allowing folding to occur later in the *trans* side of the membrane, (b) management of protein “solubility,” to prevent aggregation, (c) incorporating intrinsic signals that can tell two polypeptides apart and allow some of them to be targeted to membranes, and (d) acquisition of final native structures that satisfy all the above criteria and yet provide a sufficient gamut of structures for all cell-envelope chemistries. This “exportome non-folding problem” prior to secretion, is in a sense the inverse of the core biological “folding problem” (Dill et al., 2008), driven by the same fundamental physics principles, and is reminiscent of the behavior of IDPs (Zhou and Dunker, 2018). Undoubtedly, specific intrinsic polypeptide features allow evolution to select the combination of non-folding, solubility, targeting signals, TM crossing, and endpoint folding. These features have remained obscure. Soluble exportome polypeptides have been largely considered to be similar to cytoplasmic ones and their ability to remain non-folded and soluble and become secreted was relegated to chaperones, translocases, and N-terminal signal peptides (De Geyter et al., 2016; Tsirigotaki et al., 2017).

In contrast, our analysis of the K-12 proteome and experimental data (Chatzi et al., 2017; Sardis et al., 2017; Tsirigotaki et al., 2018), reveal an unsuspected richness of the subcellular topology structural landscape. While retaining a fundamental common wiring, the cytoplasmome and secretome are strikingly different at multiple levels. These structural differences represent adaptations in secretory mature domains, which have escaped previous scrutiny and are independent of signal peptides (that have been completely excluded from our study).

Primary sequence differences between cytoplasmome and secretome are pronounced. Secreted polypeptides are enriched in small, polar and more soluble residues, show higher disorder and rCO, have fewer and weaker hydrophobic patches and APRs, all suggestive of slower folding and dynamic native structures (Plaxco et al., 1998; Orfanoudaki et al., 2017; Tsirigotaki et al., 2018). Additionally, amino acids whose synthesis requires less ATP are used significantly more in the secretome than in the cytoplasmome (Smith and Chapman, 2010). These differences are so prevalent, that we could confidently use only a small number of them as features to tell cytoplasmome/secretome polypeptides apart with 91.5% (MatureP; Orfanoudaki et al., 2017) or 95.5% (Table 1) confidence.

Primary sequence variability drives folding kinetics and higher order organization. Forty-three percentage of the K-12 proteome is predicted to contain IDRs, of which 4% IDPs. Both parameters are particularly enriched in the secretome. We hypothesize that this adaptation serves two main purposes: firstly, it minimizes the chances of premature cytoplasmic folding, independently of the presence of any chaperone, and secondly, it optimizes TM crossing through the lipid-embedded Sec translocase in non-folded states. These notions are corroborated by experimental evidence with structural twins: even moderately disordered secreted proteins fold more slowly than their cytoplasmic counter parts (Tsirigotaki et al., 2018). Additionally, enhanced inherent disorder and flexibility may satisfy a third purpose of specific cell envelope related functions (Supplementary Table S9), e.g., binding prosthetic groups (e.g., NrfB; Clarke et al., 2007), chaperoning (Skp, Spy, SurA, PpiA, HdeA; Walton and Sousa, 2004; Burmann et al., 2013), interaction with OM proteins and conformational linkage to the IM (TonB; Sean Peacock et al., 2005), sensing stress (RcsF; Rogov et al., 2011), peptidoglycan binding and periplasm-cell surface topological transitions (Lpp; Liu et al., 2002); small molecule (Tompa et al., 2006) and colicin (Johnson et al., 2017) import and phage adsorption (DcrB; Likhacheva et al., 1996), lateral Bam opening to facilitate porin insertion (Hagan et al., 2011), curli subunits that are additionally secreted across the OM like the amyloid fiber CsgA and the CsgF lid (Raivio et al., 1999; Van Gerven et al., 2015). Disorder can also have additional relevant functions, e.g., by facilitating multiple interactions it can yield higher thermostability as in the small ribosomal subunits of *Thermus thermophilus* when compared to those of the mesophilic *E. coli* (Mallik and Kundu, 2013).

Short and long IDRs are heavily enriched in multiple protein classes (Figure 3G) and their location within the protein sequence may contribute to protein function and dynamics. In the cytoplasmome, many transcription factors have N-terminal and internal DNA binding IDRs (Lobley et al., 2007) and highly disordered ribosomal proteins, 18% of them IDPs, have terminal IDRs (Peng et al., 2014). Peripheral IM proteins are cytoplasmic proteins that can also bind to membranes on IM proteins (e.g., SecA; Tsirigotaki et al., 2017) or lipids (e.g., PspA; McDonald et al., 2017). Some peripheral IM proteins are IDPs such as those involved in cell division (e.g., ZapB; Ebersbach et al., 2008), RNA degradation (RNaseE; Callaghan et al., 2004) and the RNA chaperone ProQ (Smith et al., 2007). Several peripheral IM proteins, many involved in protein-protein interactions, contain middle (73%) or C-terminal (40%) IDRs (Papanastasiou et al., 2013). This flexibility may control substrate binding, as suggested for SspB (Wah et al., 2003). Manual search revealed that IDRs encompass substrate-binding regions and active enzyme sites, but more detailed analysis on what function these regions have is needed. More than half of the highly disordered secreted lipoproteins have disordered N-termini that might function as expandable, flexible tethers between the protein and its membrane anchor. These IDRs might play a role in targeting, stress sensing, surface exposure (Paliy et al., 2008; Zuckert, 2014) and IM-to-OM distance sensing (Asmar et al., 2017). IDR-mediated flexibility may help chaperones bind to multiple substrates (Gorovits and Horowitz, 1995; van der Lee et al., 2014). C-terminal IDRs regulate flexibility and act as an auto-inhibitory substrate mimic in SecA (Chatzi et al., 2014), cytoplasmic foldase DnaK (Smock et al., 2011) and several flagellar and pathogenic Type 3 secretion chaperones (Chen et al., 2013; Little and Coombes, 2018). Internal IDRs in OM proteins (e.g., OmpA, OmpC, BamA) presumably control pore flexibility for *trans*-membrane transport. OM protein disorder might help them bind to chaperones and be targeted to the OM (Paliy et al., 2008), stress sensing, surface exposure (Zuckert, 2014) and IM-to-OM distance sensing (Asmar et al., 2017). IM proteins, the least IDP-rich group, also contain regions of enhanced disorder with specific functions: ZipA to dock to the cell division ring (Vicente and Rico, 2006) and to YtfB, DedD, DamX, FtsN (Gerding et al., 2009); TatB to mediate folded protein export (Patel et al., 2014); RseA to sense envelope stress and bind to sigmaE (De Las Penas et al., 1997); the FliF flagellar ring to allow rotational motion (Grunenfelder et al., 2003); FtsH and its modulator HflK to degrade different IM proteins (Asahara et al., 2000).

Given the high disorder and slower folding of the secretome (Tsirigotaki et al., 2018), it is unsurprising that specific evolutionary adaptations are needed to secure that its polypeptides can acquire their final folded states. Enhanced secretome native state thermostability (Figure 2B, VI; Leuenberger et al., 2017; Mateus et al., 2018) may compensate for the elevated dynamics of the folding intermediates, while stability of native OM proteins comes from the lipid-embedded state (Lessen et al., 2018). In addition, up to a third of native states in secretome polypeptides are stabilized by disulfides (Supplementary Table S3), oligomerization (e.g., HdeA, PhoA, Spy, CsgA, Lpp), metal ion binding (e.g., Ca^2+^, glucose binding protein; Herman et al., 2005) and prosthetic groups (e.g., the cytochrome c-type protein NrfB; Clarke et al., 2007) and many other solutions (De Geyter et al., 2016).

Structural folds in final native states also tell the secretome and cytoplasmome apart. The secretome, both soluble and OM proteins, is β-rich. It is also reduced in α/β folds, that are actually depleted in its unique domains, suggesting active evolutionary pressure. The SecY translocase channel “scans-and-sorts to lipid” exported chains with helical hydrophobic segments (Tsirigotaki et al., 2017). Extended β strands, as in the OM proteins, were selected because they escape SecY, due to the alternate planes of their side chains and reduced hydrophobicity. Enhanced disorder, shorter and more dynamic hydrophobic patches, altered APRs/gatekeepers and altered amino-acid content (Figure 2) may all contribute to optimal “secretability” and/or selectively acquiring specific folds. Even the soluble secretome may have been selected to avoid many α-helices with hydrophobic faces that could hamper SecY passage. Many secretome α proteins are short, hydrophilic and highly flexible (e.g., the chaperone Spy, extracellular YebF, the peptidoglycan binding and trimerizing Lpp; Tsirigotaki et al., 2018).

Two thirds of the folds in the secretome are frequent and shared with the cytoplasmome (Figure 4D,E). The secretome has far fewer rare and unique domains suggesting that only some of these may have been adaptable to the process of secretion. Perhaps only particular secretome folds could be selected in response to export-specific requirements, function or folding in the cell envelope. Unique secretome folds may provide specific functionalities that are only relevant to the cell envelope such as maintenance of OM structure (e.g., lipocalin-fold b.60 in lipoprotein Blc), stress response (e.g., “trypsin-like serine protease”-fold b.47 in DegP, DegS, and DegQ) and peptidoglycan biosynthesis (e.g., “Penicillin-binding protein associated domain”-fold b.105 in DacA, DacC, and DacD; Supplementary Table S3). Collectively, how domain architectures are distributed across the cytoplasmome and the secretome appears non-random and seems actively selected.

Chaperones acting close to or at the ribosome can be important regulators for cytoplasmome abd secretome traffic. These interactions confer a “positive chromatography” effect, sequester exported proteins out of cytoplasmic circulation, reduce the danger of highly hydrophobic, exported molecules being inadvertently released in the cytoplasm (Huber et al., 2005) and pilot them to membrane-embedded translocases (Figure 7A, steps I and II). Exportome proteins that cannot be secreted (e.g., during stress) might interact with cytoplasmic chaperones acting as holdases (Supplementary Table S17 and Supplementary Figure S7D), without any appreciable folding (Figure 7F). IM proteins also interact with SecA, perhaps corroborating its proposed involvement in co-translational IM protein integration into the IM (Figure 7A; Wang et al., 2017). In contrast, cytoplasmic polypeptides that escape this scrutiny, diffuse into the cytoplasm and fold, with or without foldases (Figure 7A, step III; Supplementary Table S17).

These observations all lend support to a fundamental concept: the dominant, inherent nature of polypeptides to fold fast or to remain disordered unaided (Anfinsen, 1973; Dunker et al., 2013; Sardis et al., 2017; Tsirigotaki et al., 2018). Primarily intrinsic, and additional extrinsic, features lead to cytoplasmome/secretome differences (Figure 7F). Intrinsic features maintain the non-folded secretome soluble and translocation-competent in the cytoplasm, and provide targeting export signals recognized by chaperones or the translocase. Enhanced flexibility during cytoplasmic transit facilitates secretion and prevents unwanted premature folding. Signal peptides can partially delay folding for structures whose mature domains could not be directly manipulated (like the α/β maltose binding protein; Beena et al., 2004), but are a less robust and weaker solution than intrinsic disorder (Tsirigotaki et al., 2018). The secretome folds in the cell envelope and beyond, into a small repertoire of folds that retain extreme disorder and flexibility (Figure 7F, right), presumably reflecting functional adaptations to cell envelope specific chemistries.

## Summary

**FIGURE 8:**
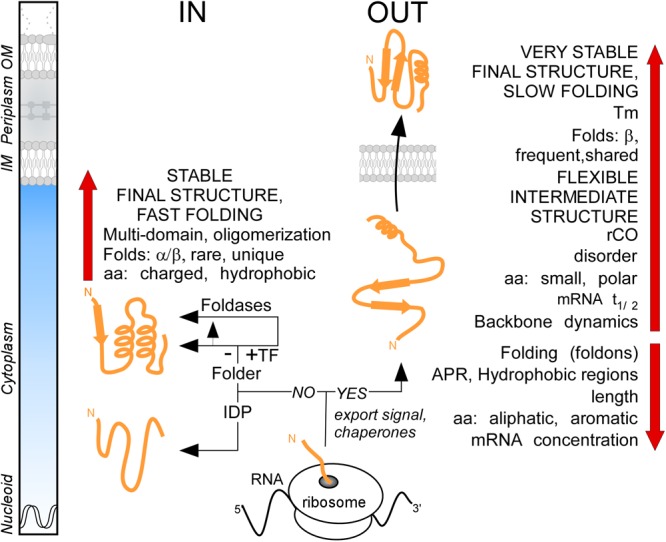
Summary. Cartoon representation of protein synthesis and folding at final destinations in K-12 (IN: Cytoplasmome; OUT: Secretome). Important factors and check-points influencing this process are summarized. See text for details. aa, amino acids; APR, aggregation prone region; IDP, intrinsically disordered protein; IM, inner membrane; OM, outer membrane; rCO, relative contact order; TF, trigger factor; Tm, melting temperature.

It was generally thought that the major distinction between cytoplasmic and secreted proteins is the presence of the signal peptide in the latter. By analyzing the proteome of *E. coli* K-12, we demonstrate that these two protein groups have distinct characteristics in terms of primary amino acid content that then leads to different folding propensities, secondary structure preferences, degrees of disorder and structural folds (Figure 8). We found that the secretome displays unusually enhanced flexibility, slow folding and looser structures overall (Figure 8). We hypothesize that these adaptations avoid premature folding in the cytoplasm, optimize lipid bilayer crossing and facilitate cell envelope specific chemistries and interactions. The unique combination of these features reveals new insights of protein evolution and has wide implications on the structural diversity and evolution of modern proteomes.

## Data Availability

The datasets generated and analyzed during the current study are included in this published article, its Supplementary Information Files and can be found in the STEPdb2.0 database (http://stepdb.eu/).

## Author Contributions

ML and RR collected, curated, and analyzed most of the data. WV, AT, JDG, KT, BY, and VZ collected, curated, and analyzed the data sub-sets. ML, RR, WV, IT, JS, and FR performed the bioinformatics analyses. MK and E-PT managed and updated the STEPdb. ML and AE wrote the manuscript with contributions from RR, AT, WV, IT, JS, VZ, SK, and JDG. AE conceived and managed the study. All authors reviewed the final version of the manuscript.

## Conflict of Interest Statement

IT is a consultant for company Gnosis Data Analysis PC. The remaining authors declare that the research was conducted in the absence of any commercial or financial relationships that could be construed as a potential conflict of interest.

## Footnotes

1 http://stepdb.eu

## Abbreviations

APR: aggregation prone region
CO: contact order
IDP: intrinsically disordered protein
IDR: intrinsically disordered region
IFP: intrinsically flexible protein
IM: inner membrane
OM: outer membrane
rCO: relative contact order
SRP: signal recognition particle
TF: trigger factor
TM: transmembrane
