# Supplementary material for: Structural Basis of the Subcellular Topology Landscape of Escherichia coli
Source: Front Microbiol. 2019 Jul 24;10:1670. doi: 10.3389/fmicb.2019.01670 (PMC6677119; doi:10.3389/fmicb.2019.01670)
Supplement: Supplementary file 2 [file Data_Sheet_1.docx]

**Structural basis of the subcellular topology landscape of *Escherichia coli***

Supplementary Material

**Maria S. Loos^1^**^#^**, Reshmi Ramakrishnan^1,2^**^#^**, Wim Vranken^3,4^, Alexandra Tsirigotaki^1^, Evrydiki-Pandora Tsare^5^, Valentina Zorzini^1^, Jozefien De Geyter^1^, Biao Yuan^1^, Ioannis Tsamardinos^6,7^, Maria Klappa^5^, Joost Schymkowitz^2^, Frederic Rousseau^2^, Spyridoula Karamanou^1^, and Anastassios Economou^1,6*^**

^1^ Laboratory of Molecular Bacteriology, Rega Institute, KU Leuven Department of Microbiology and Immunology, Herestraat 49, B-3000 Leuven, Belgium

^2^ VIB-KU Leuven Center for Brain & Disease Research and VIB Switch Laboratory, Department for Cellular and Molecular Medicine, KU Leuven, Herestraat 49, 3000, Leuven, Belgium

^3^ Interuniversity Institute of Bioinformatics in Brussels, ULB-VUB, La Plaine Campus, Triomflaan, C building, CP 263, 1050 Brussels, Belgium

^4^ Structural Biology Brussels, Vrije Universiteit Brussel and Center for Structural Biology, VIB, Pleinlaan 2, Brussels 1050, Belgium

^5^ Metabolic Engineering & Systems Biology Laboratory, Institute of Chemical Engineering Sciences, Foundation for Research and Technology-Hellas (FORTH/ICE-HT), Patras, Greece

^6^ Gnosis Data Analysis PC, Palaiokapa 64, 71305, Heraklion, Crete, Greece

^7^ Department of Computer Science, University of Crete, Voutes Campus, GR-70013 Heraklion, Crete, Greece

^#^ Equal contribution

* Correspondence:

Anastassios Economou

[tassos.economou@kuleuven.be](mailto:tassos.economou@kuleuven.be)

**Running Title:** K-12 topology landscape

**Keywords: Protein secretion; Cytoplasmome; Protein Disorder; Protein Domains; Protein folding; Protein subcellular localization; Protein targeting; Secretome.**

**List of abbreviations:**

APR: Aggregation Prone Region; CBS: Client Binding Sites; IDP: intrinsically disordered protein; IDR: intrinsically disordered region; IM: Inner Membrane; OM: Outer Membrane; PDB: Protein Data Bank; PMP: Protein Model Portal; RPKM: Reads Per Kilobase of transcript, per Million mapped reads; rCO: relative Contact Order; SMR: SWISS-MODEL Repository; SRP: signal recognition particle; TAP: Tandem Affinity Purification; TBM: Template based model; TF: trigger factor; Tm: melting temperature; TM: transmembrane.

# ****1 Table of contents****

# 2 Supplementary Figures and Tables

# 2.1 Supplementary Figures

**Figure S1** Longitudinally positioned proteins (related to Figure 1)

**Figure S2** Comparison between cytoplasmic and exported proteins (related to Figure 2)

**Figure S3** Characteristics of global and local features of cytoplasmic, IM and secreted proteins (related to Figure 2 and 3)

**Figure S4** TMHMM, Hydrophobicity and IUpred2 predictions for SecG protein (related to Figure 2 and 3)

**Figure S5** Folds in the cytoplasmome compared to the exportome (related to Figure 4)

**Figure S6** Distribution of N to C termini distances (related to Table S3)

**Figure S7** Interactors of ribosome bound and soluble chaperones as determined using ribosome profiling and TAP-tag techniques (related to Figure 7)

**Figure S8** Workflow *E. coli* K-12 structures and models (related to Table S7 and 8)

# 2.2 Supplementary Tables

**Table S1 (XLS)** Protein subcellular locations summary

**Table S2 (XLS)** Changes and corrections in annotations to STEPdb 2.0

**Table S3** **(XLS)** Features of K-12 proteins

**Table S4 (XLS)** K-12 longitudinal positioning of proteins

**Table S5 (XLS)** K-12 Chaperonome

**Table S6 (XLS)** K-12 Proteaseome

**Table S7 (XLS)** Pipeline for K-12 proteome structure assignment

**Table S8 (XLS)** K-12 Proteome structures list

**Table S9 (XLS)** Foldon regions, IDRs, APRs and hydrophobic regions in the K-12 proteome

**Table S10 (XLS)** Early Foldon regions in K-12 proteins

**Table S11 (XLS)** Intrinsically Disordered Regions in K-12 proteins

**Table S12 (XLS)** Hydrophobic regions in K-12 proteins

**Table S13 (XLS)** Aggregation Prone Regions in K-12 proteins

**Table S14 (XLS)** K-12 structural and functional folds

**Table S15 (XLS)** K-12 change in protein abundance across various growth conditions

**Table S16 (XLS)** K-12 SRP, TF and SecA interactors in K-12

**Table S17 (XLS)** Soluble chaperone interactors in K-12

**Table S18** Features that differentiate cytoplasmome from secretome selected by machine learning

# 3 Supplementary Materials and Methods and Results

Additional bacterial strains

Universal protein names

Cataloguing a list of the proteostatic machinery: chaperones and folding factors

Cataloguing a list of the proteostatic machinery: proteases

Protein characteristics analysis

Cataloguing a comprehensive list of the K-12 structural proteome

Structural data analysis

Analysis of the disulfideome

Change in protein abundance

Treatment of ribosome profiling data

Cataloguing soluble chaperone interactors

Statistical analysis

**4 References**

# 2 Supplementary Figures and Tables

# 2.1 Supplementary Figures


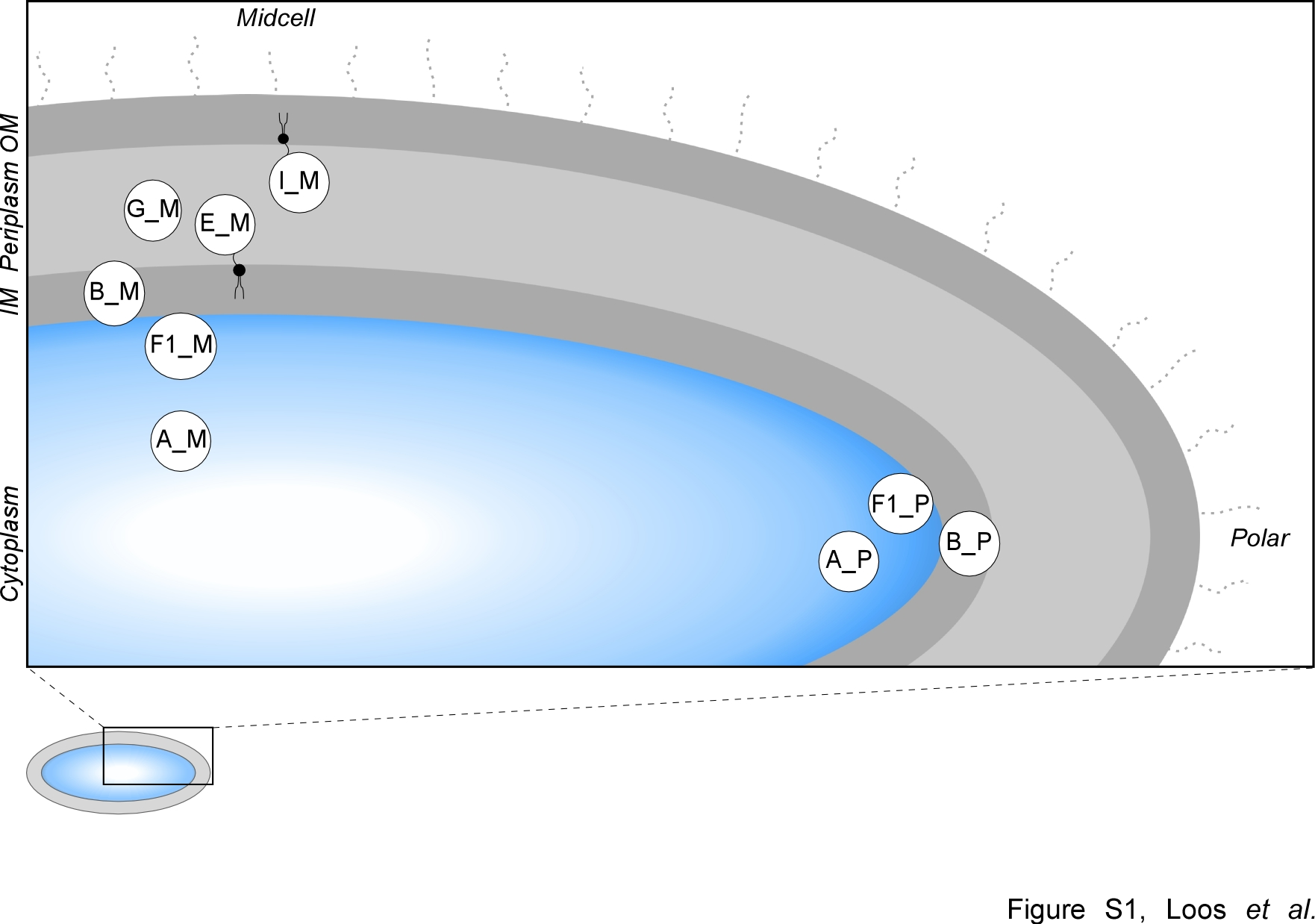


**Figure S1 Longitudinally positioned proteins (related to Figure 1)**

Cartoon representation of proteins positioned longitudinally to either the midcell or polar positions. Apart from their localization in different cellular compartments (Fig. 1A), several peripheral IM or exportome proteins with important cellular functions can accumulate at specific sites of the plasma membrane in the longitudinal axis of the cell (Table S4). This “longitudinal positioning” includes positions of polar (STEPdb symbol followed by “_P”) and mid-cell localization (STEPdb symbol followed by “_M”). Examples include the polarly located Tsr (Liberman *et al.* 2004), and chemotaxis proteins CheW and CheA (Maddock and Shapiro 1993; Shapiro *et al.* 2009) and the midcell located cell division factors MinE, MinC and MinD (Dworkin 2009; Shapiro *et al.* 2009; Shih and Zheng 2013). Misfolded proteins are often also stored at the bacterial poles in inclusion bodies (Laloux and Jacobs-Wagner 2014). Equatorially positioned proteins include *e.g.* cytoplasmic FtsA, FtsZ and periplasmic FtsQ, FtsB that form the division ring (see also the interactive Cell Atlas in STEPdb 2.0 (<http://stepdb.eu/cell_cartoon.php>); (Hale and de Boer 1999; Dworkin 2009; Glas *et al.* 2015)), the cell division proteins SlmA, ZapA and ZipA (Rudner and Losick 2010) and the cell wall synthesis protein MurG (Dworkin 2009). Another protein class with extended filamentous structures and longitudinal positioning are actin-like MreB filaments (Errington 2015). MreB is essential for the bacterial rod shape and its filaments align along the greatest principal membrane curvature (Fig. 2A; (Hussain *et al.* 2018)).


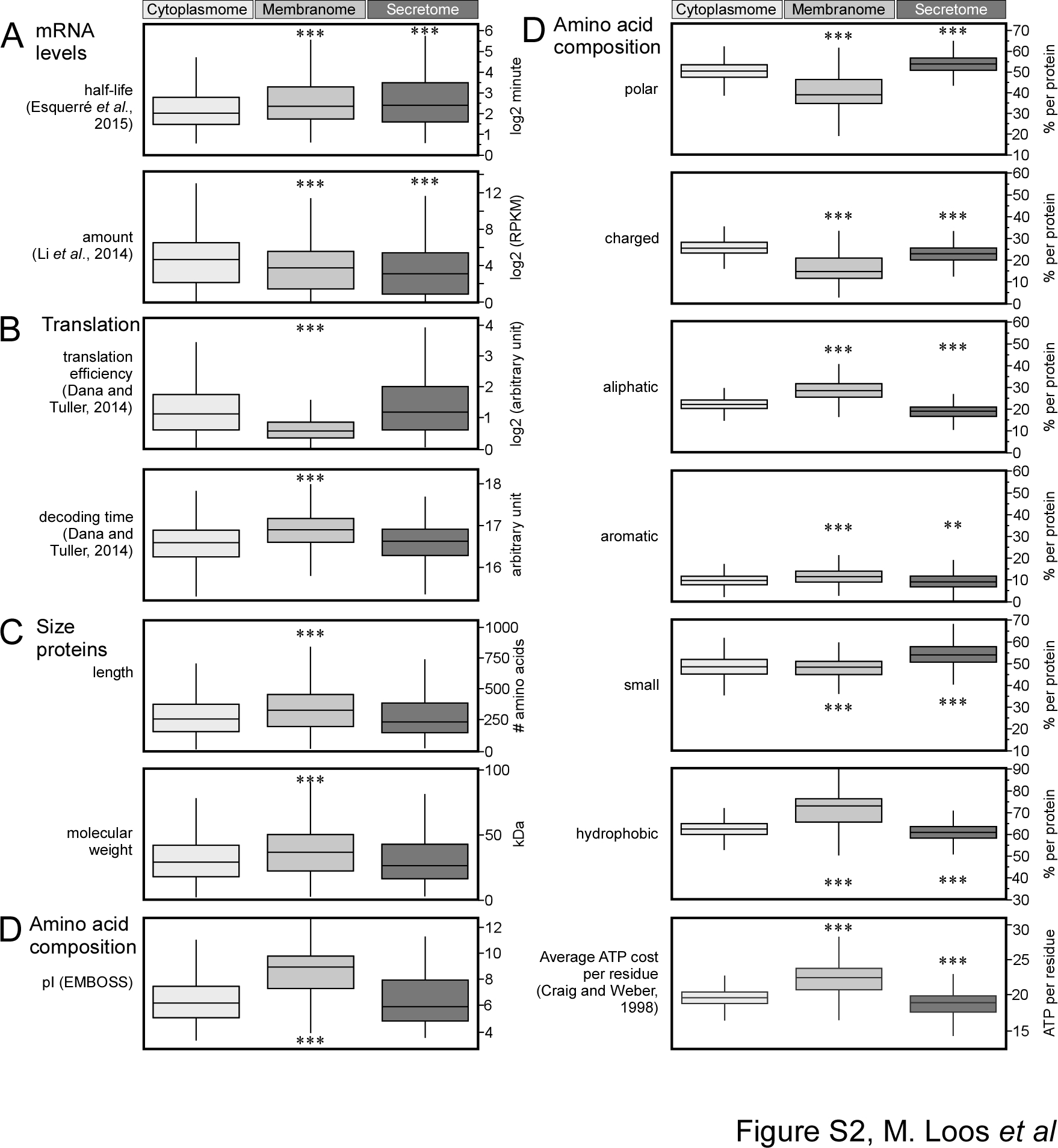

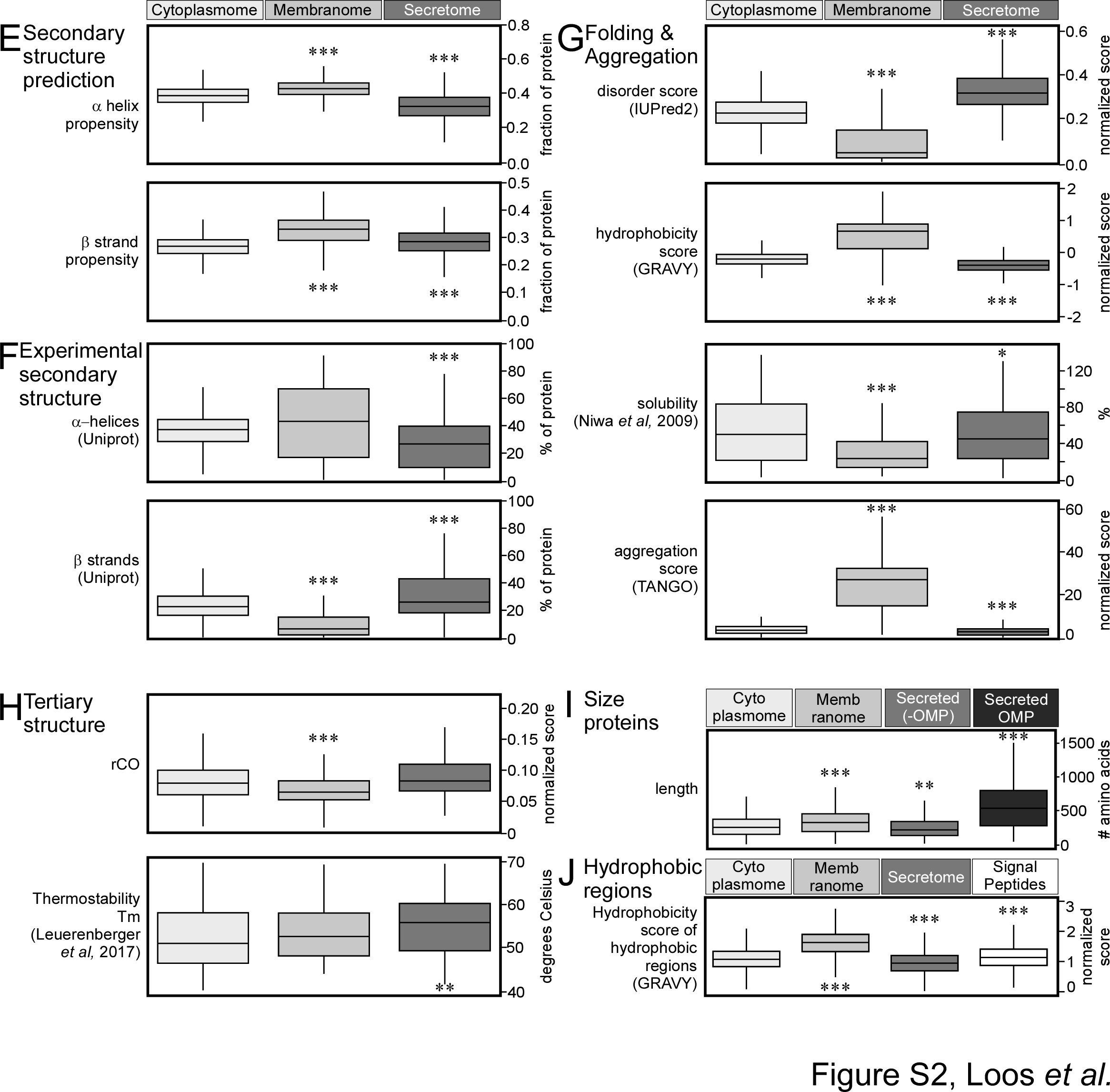


**Figure S2 Comparison between cytoplasmic and exported proteins (related to Figure** **2)**

Distributions of intrinsic features of cytoplasmome (light grey), membranome (grey) and secretome (OM proteins included except in panel I; dark grey) shown in box plots. **A.** mRNA characteristics (adapted from Esquerré *et al* (Esquerre *et al.* 2015) and Li *et al* (Li *et al.* 2014)). **B.** Translation efficiency and decoding times (calculated as suggested from Dana and Tuller (Dana and Tuller 2014); see Experimental procedures). **C.** Length and molecular weight of proteins as obtained from Uniprot (Dimmer *et al.* 2012). **D.** Amino acid composition (as described in Experimental Procedures), calculated using with in-house written scripts as described in Supplemental Experimental Procedures. **E.** Predicted secondary structure propensity, as described previously (Cilia *et al.* 2013; Raimondi *et al.* 2017). **F.** Experimental secondary structure content, as obtained from Uniprot (Dimmer *et al.* 2012). **G.** Disorder (IUPred2; (Meszaros *et al.* 2018)) and aggregation (TANGO; (Fernandez-Escamilla *et al.* 2004)) predictions, protein solubility (adapted from Niwa *et al* (Niwa *et al.* 2009)) and hydrophobic predictions (GRAVY; (Kyte and Doolittle 1982)) obtained as described in Experimental procedures. **H.** Tertiary structure characteristics of melting temperature (Tm; adapted from Leuenberger *et al* (Leuenberger *et al.* 2017)) and calculated relative Contact order (rCO; as described in Plaxco *et al* (Plaxco *et al.* 1998)). **I.** Length of proteins (Uniprot; (Dimmer *et al.* 2012)), OM proteins analyzed separately. **J.** Only hydrophobic regions predicted from different topological groups and signal peptide sequences predictions for hydrophobicity (by GRAVY tool). Statistical analysis was done using Kruskal-Wallis and Fisher's exact tests: * = p<0.05; ** = p<0.01; *** = p<0.001 for the comparison between cytoplasmome and other topology groups (significance indicated on top of the latter; in case of no significant difference, the stars are not indicated). Abbreviations: rCO = relative Contact Order; OMP = Outer Membrane Proteins; RPKM = Reads Per Kilobase of transcript, per Million mapped reads; Tm = melting temperature.


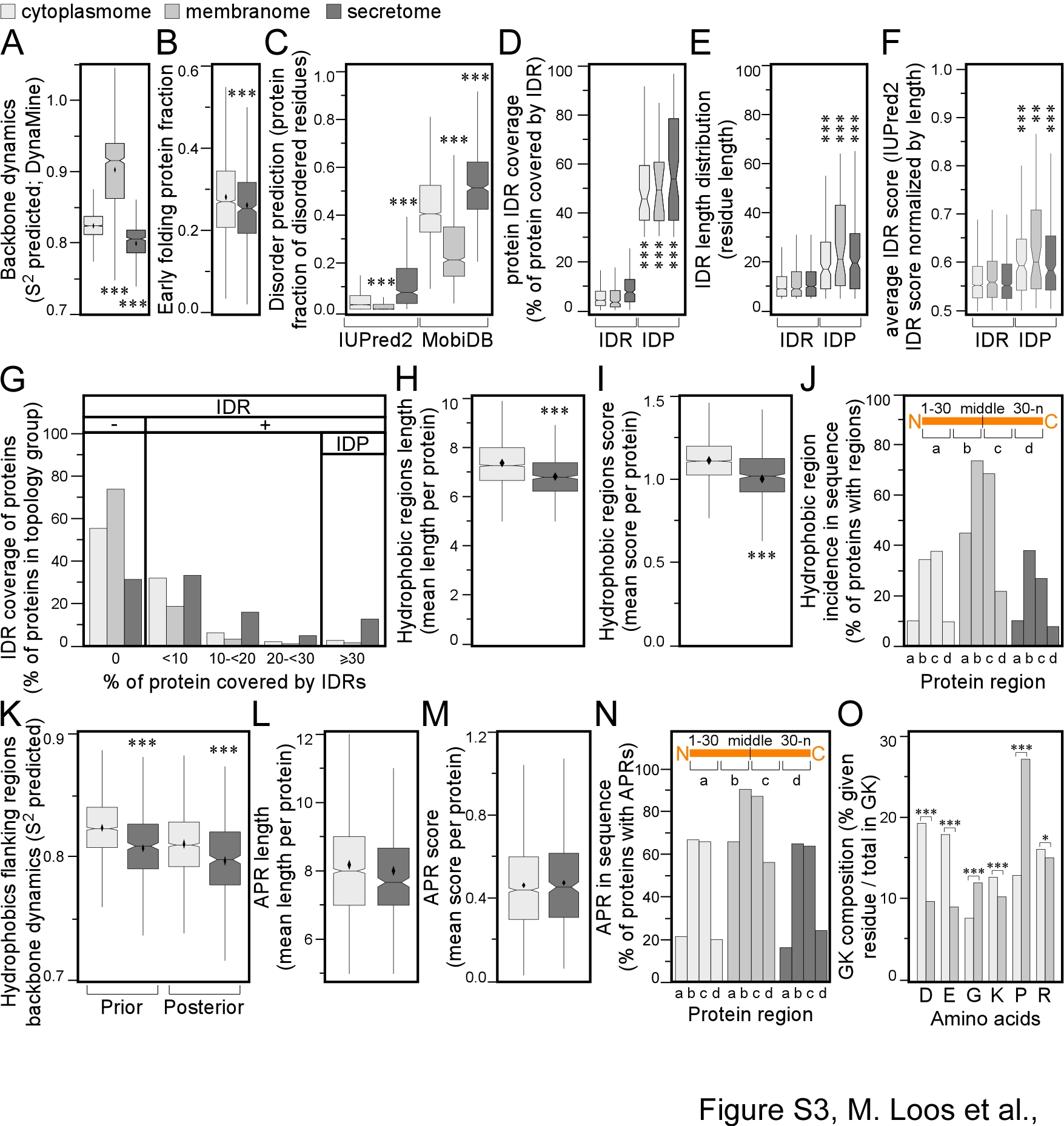


**Figure S3 Characteristics of global and local features of cytoplasmic, IM and secreted proteins (related to Figure 2 and 3)**

**A.** Backbone dynamics predictions (DynaMine) as described previously (Cilia *et al.* 2013). Although the tool was not trained on the membrane proteins, we found that membranome is more rigid group. Low S2 predicted values indicate increased propensity for highly dynamic backbones. **B.** Early folding predictions (EFoldMine) as described previously (Raimondi *et al.* 2017). Early folding fraction is calculated as total amino acids predicted to fold early in a protein per total number of amino acids in this protein. **C.** Fraction of amino acids that are disordered according to IUPred2 (score > 0.5; left) and MobiDB (consensus score; right) for each protein. We found multiple small/short proteins that are completely disordered, which contributes to higher average score for MobiDB. For panels A-C, statistical analysis was done using Kruskal-Wallis and Fisher's exact tests: * = p<0.05; ** = p<0.01; *** = p<0.001 for the comparison between cytoplasmome and other topology groups (significance indicated on top of the latter). **D.** Protein coverage by IDRs for proteins with IDRs (left) or IDPs (right) for different topologies. **E.** Average IDR length per protein for proteins with IDRs (left) or IDPs (right) for different topologies. **F.** Average IDR length per protein for proteins with IDRs (left) or IDPs (right) for different topologies. For panels D-F, statistical analysis was done using Kruskal-Wallis Test or Fisher's exact tests: ** = p<0.01; *** = p<0.001 for the comparison between proteins with IDRs (with IDR) and IDPs (IDP) for each topology group (significance indicated on top of the latter). **G.** Sequence coverage by IDRs presented in percentage groups on the x-axis. IDR covers proteins having either no (-) IDRs or ≥1 IDR (+) and IDP (+) includes only the IDPs. **H.** Average length of hydrophobic regions per protein. **I.** Average score of hydrophobic regions per protein. **J.** Percentage of proteins with hydrophobic region in each topology group that have the hydrophobic regions in the first 30 residues of the N-terminus (a), last 30 residues of the C-terminus (d) or in the remaining middle region (excluding regions a and d) divided in two equal halves (b and c). See cartoon at the top of the graph for schematic representation. **K.** Backbone dynamics predictions (DynaMine) for the flanking regions N-terminally (5 amino acids before the start of the hydrophobic region; Prior) and C-terminal (5 amino acids after the hydrophobic region; Posterior), as described previously (Cilia *et al.* 2013). **L.** Average length of APRs per protein (no significant difference found; (Fernandez-Escamilla *et al.* 2004)). **M.** Average score of APRs per protein (no significant difference found). **N.** Percentage of proteins with APRs in each topology group that have the APRs in same regions as described in panel J (see cartoon at the top of the graph). **O.** Relative frequently used amino acids (D: Aspartic Acid; E: Glutamic Acid; G: Glycine; K: Lysine; P: Proline; R: Arginine) in gatekeepers of proteins in different subcellular topology groups. Statistical analysis for panels H-I, K-M and O was done using Kruskal-Wallis and Fisher's exact tests comparing cytoplasmome to other topology groups: * = p<0.05; ** = p<0.01; *** = p<0.001. Abbreviations: aa = amino acid; APR = Aggregation Prone Region; GK = Gatekeeper; IDP = intrinsically disordered protein; IDR = intrinsically disordered region.


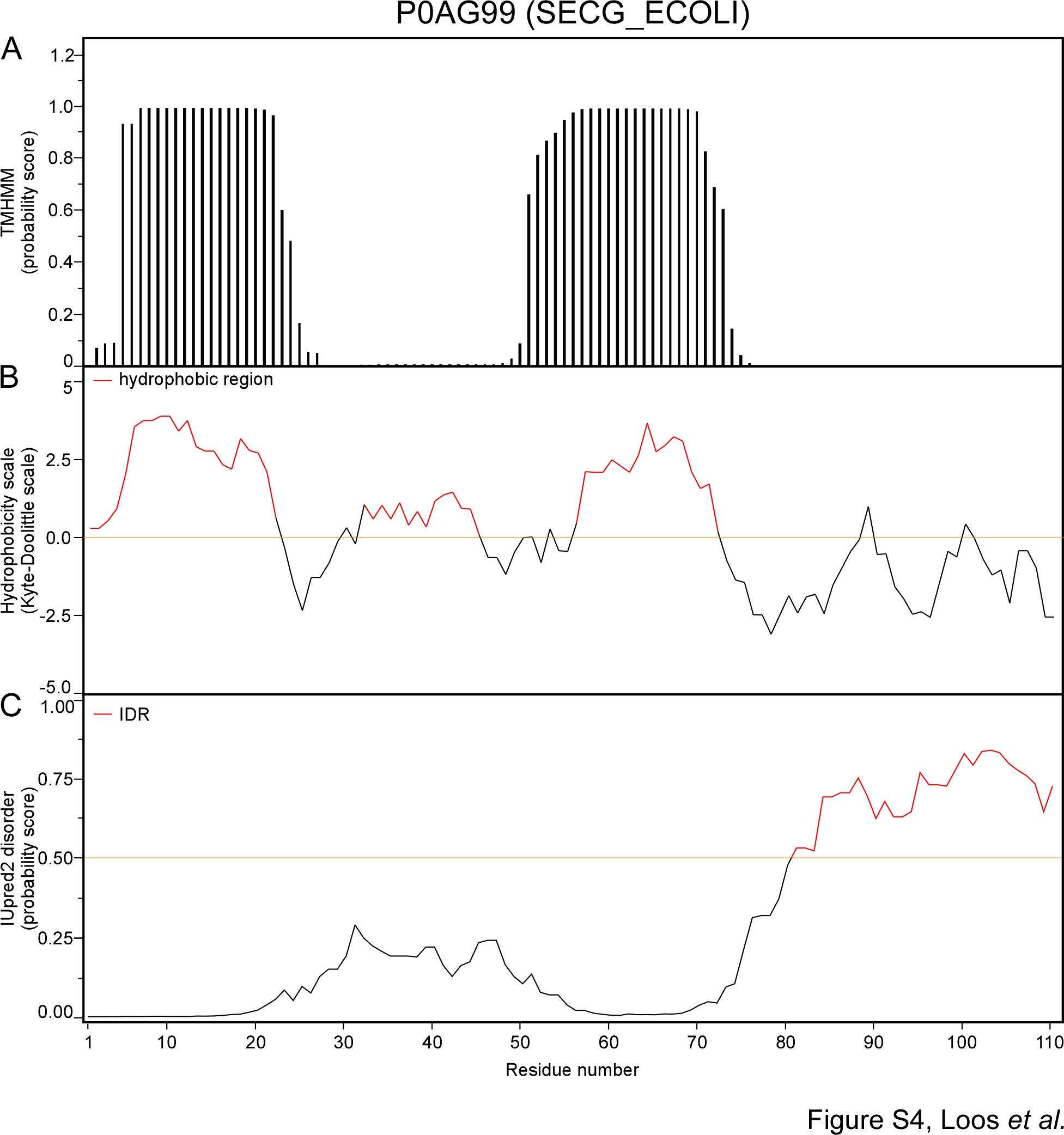


**Figure S4 TMHMM, Hydrophobicity and IUPred2 predictions for SecG protein (related to Figure 2 and 3)**

**A.** Predictions of TM domain probability using TMHMM tool (Krogh *et al.* 2001). **B.** Predictions of hydrophobicity using Kyte-Doolittle scale (GRAVY; (Kyte and Doolittle 1982)), based on sliding window (see Experimental procedures). Regions depicted in red are predicted hydrophobic regions. **C.** Predictions of disorder using the IUPred2 tool. Region above the orange line is the IDR (see Experimental procedures). Abbreviations: IDR = intrinsically disordered region; TM = transmembrane.


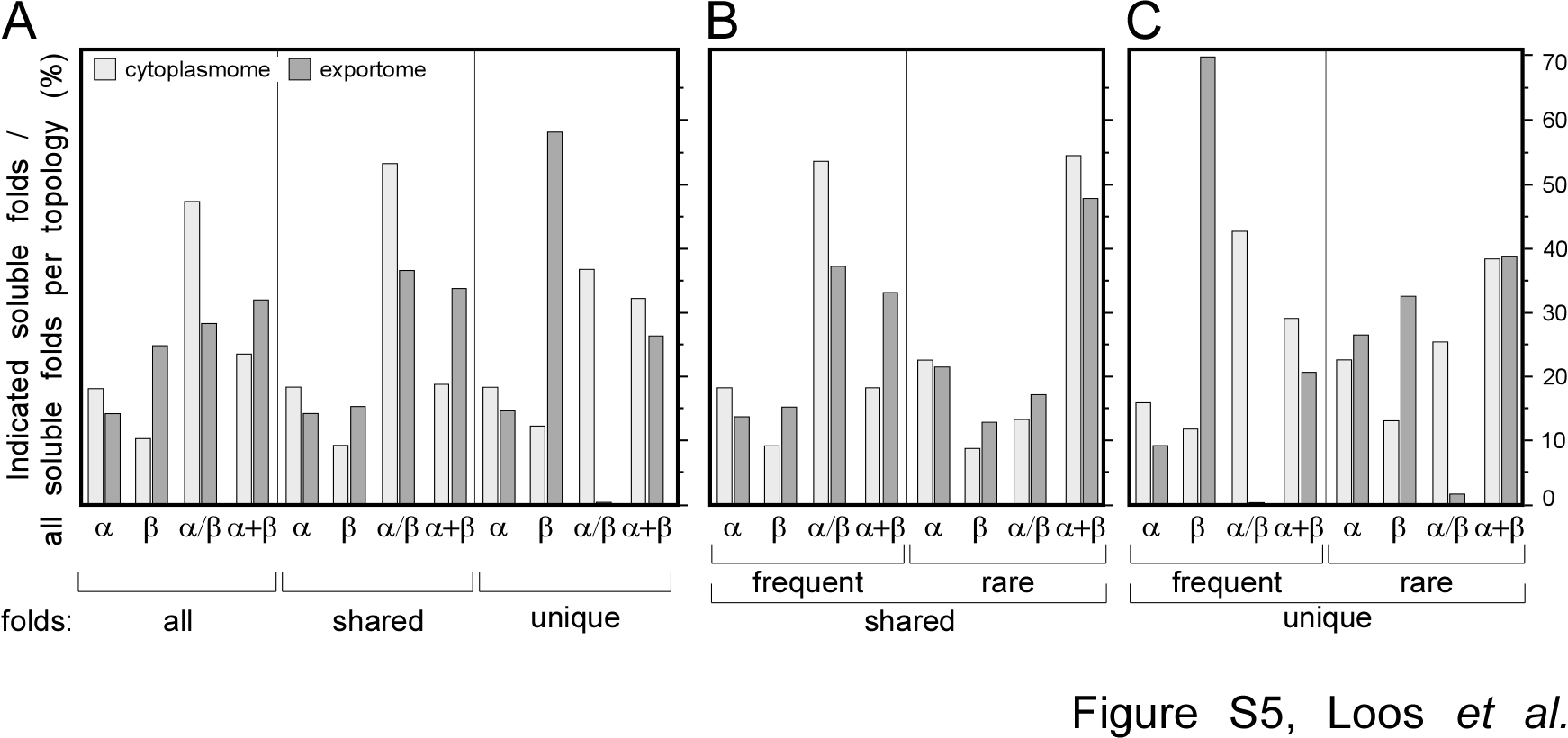


**Figure S5 Folds in the cytoplasmome compared to the exportome (related to Figure 4)**

**A.** Indicated soluble fold classes in cytoplasmic and exported proteins (Wilson *et al.* 2009). On the Left: all soluble folds in each topology group; middle: unique and right: shared folds between the two topology groups. Unique (**B.**) and shared folds (**C.**) are divided in rare and frequent folds and plotted for cytoplasmome and exportome in α, β, α/β and α+β classes.


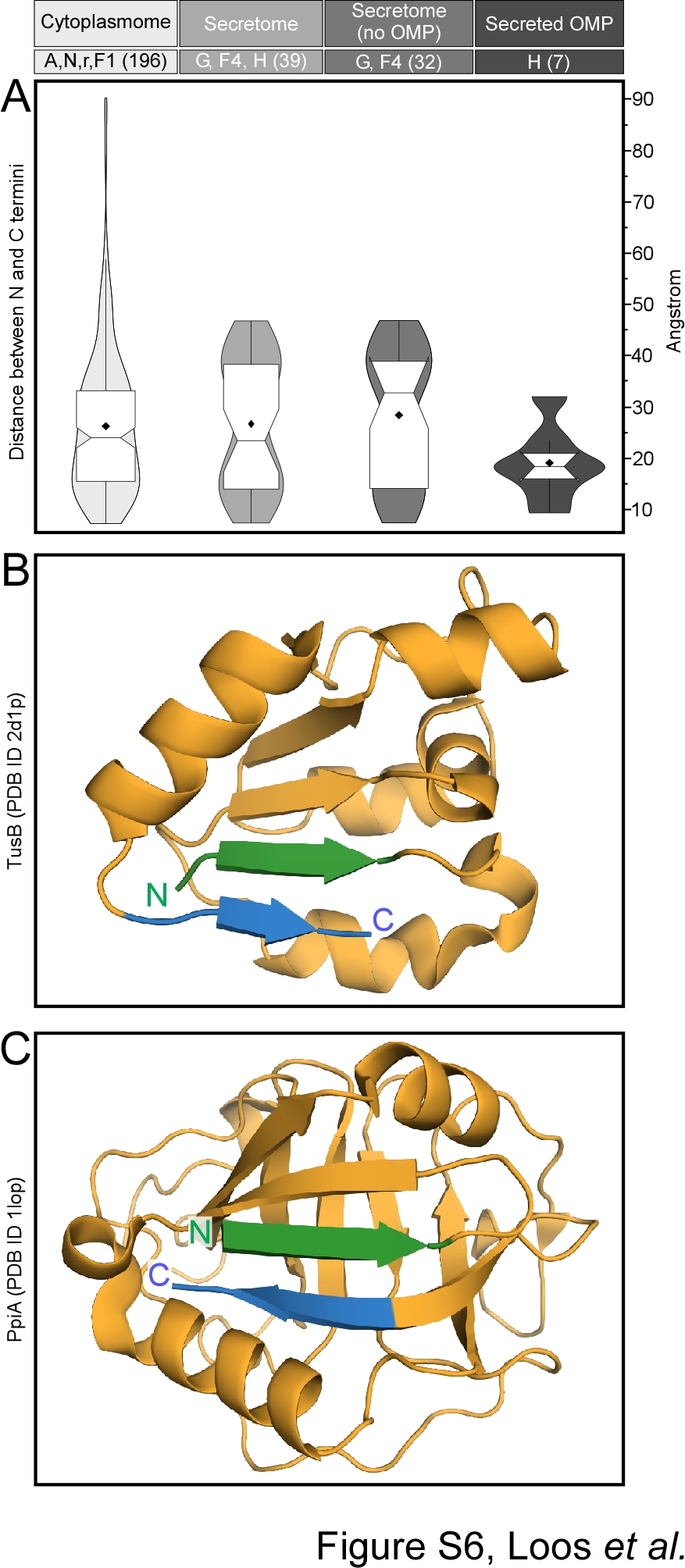


**Figure S6 Distribution of N to C termini distances (related to Table S3)**

**A.** Experimentally solved structures with 100% coverage (235 PDB structures) of cytoplasmic and secreted 1-fold proteins (containing only one annotated fold (Wilson *et al.* 2009)) were used to measure the distance between the first and the last 5 amino acids in the structure. The distribution of these distances are shown using violin plots for cytoplasmic proteins (196 structures), all secreted proteins (39 structures), secreted proteins without OM proteins (32 structures) and OM proteins separately (7 structures). Violin plots show the distribution of the data in grey and the boxplot in white, median in the middle line and the mean is represented by a black rhombus. **B.** Structure of cytoplasmic protein TusB (reference structure PDB ID 2d1p) with 5 N-terminal amino acids coloured green and 5 C-terminal amino acids coloured blue. **C.** Structure of periplasmic protein PpiA (reference structure PDB ID 1lop) with 5 N-terminal mature domain amino acids coloured green and 5 C-terminal amino acids coloured blue. Abbreviations: OMP = Outer Membrane Proteins.


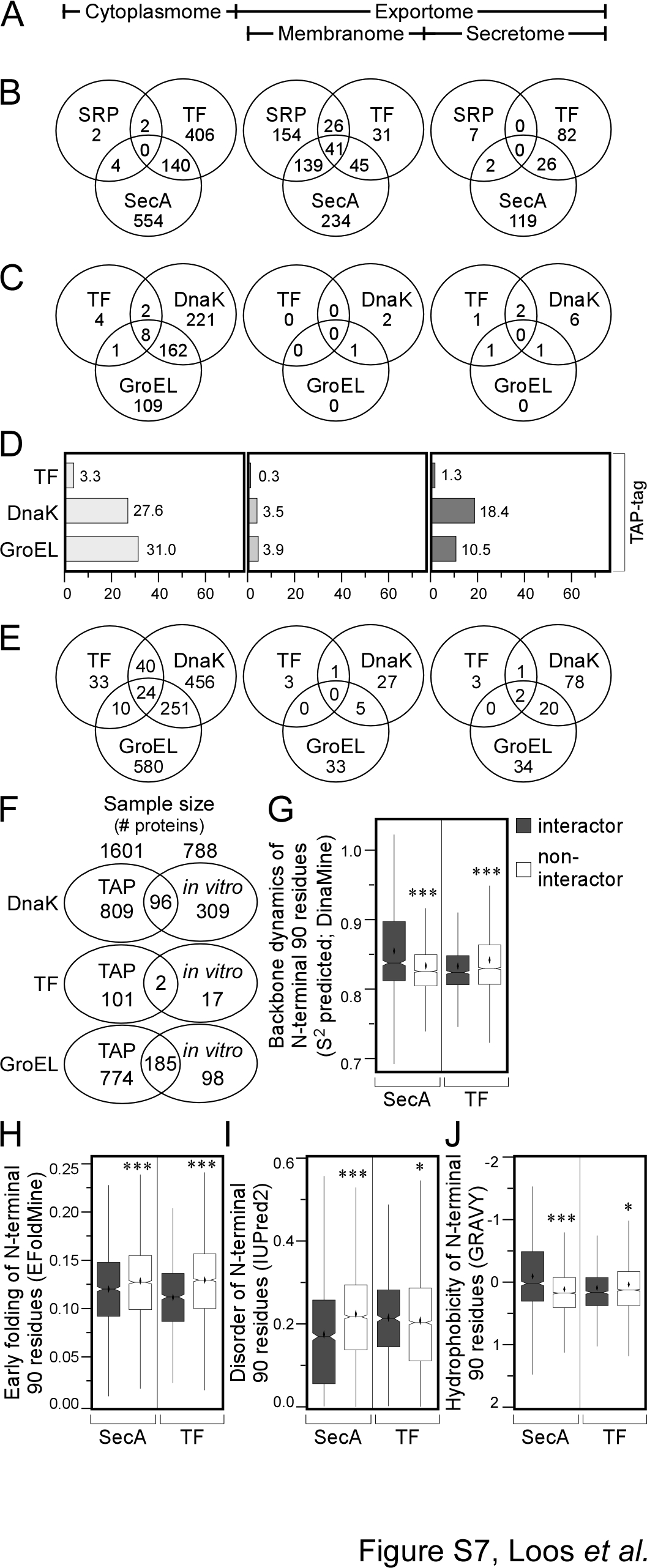


**Figure S7 Interactors of ribosome bound and soluble chaperones as determined using ribosome profiling and TAP-tag techniques (related to Figure 7)**

**A.** Schematic representation of the subcellular protein distribution in *E. coli* K-12 based. Light grey: cytoplasmome (soluble peripheral IM proteins included). Grey: membranome. Dark grey: secretome. **B.** Venn diagram representation of the overlapping nascent interactomes between SRP, TF and SecA in each subcellular localization class (left: cytoplasmome, middle: membranome, right: secretome). **C.** Venn diagram representation of the overlapping interactomes between TF, DnaK and GroEL derived from *in vitro* reconstitution method (Niwa *et al.* 2012) in each subcellular localization class as described in panel A. **D.** Interactomes of TF, DnaK and GroEL in each subcellular localization class, as identified by TAP-tag. **E.** Venn diagram representation of the overlapping interactomes between TF, DnaK and GroEL derived from TAP-tag methods in each subcellular localization class as described in panel A. **F.** Venn diagram representation of the overlapping interactomes between TAP-tag and *in vitro* reconstitution methods. **G.** Backbone dynamics (DynaMine; (Cilia *et al.* 2013)) of N-terminal 90 residues of proteins that were found to interact with SecA (left, interactors) or TF (right, interactors) with ribosome profiling or not (non-interactors). **H.** Early folding probability score predicted (EFoldMine; (Raimondi *et al.* 2017)) of N-terminal 90 residues of interactors and non-interactors of SecA and TF, respectively. **I.** Disorder prediction by (IUPred2; score normalized by length) of N-terminal 90 residues of interactors and non-interactors of SecA and TF, respectively. **J.** Hydrophobicity score (GRAVY score normalized by length; (Kyte and Doolittle 1982)) of N-terminal 90 residues of interactors and non-interactors of SecA and TF, respectively. Statistical analysis for panels G-J was done using Kruskal-Wallis and Fisher's exact tests comparing interactors and non-interactors (significance in noted on the latter): * = p<0.05; *** = p<0.001. Abbreviations: IM = Inner Membrane; TAP = Tandem Affinity Purification; TF = Trigger Factor.


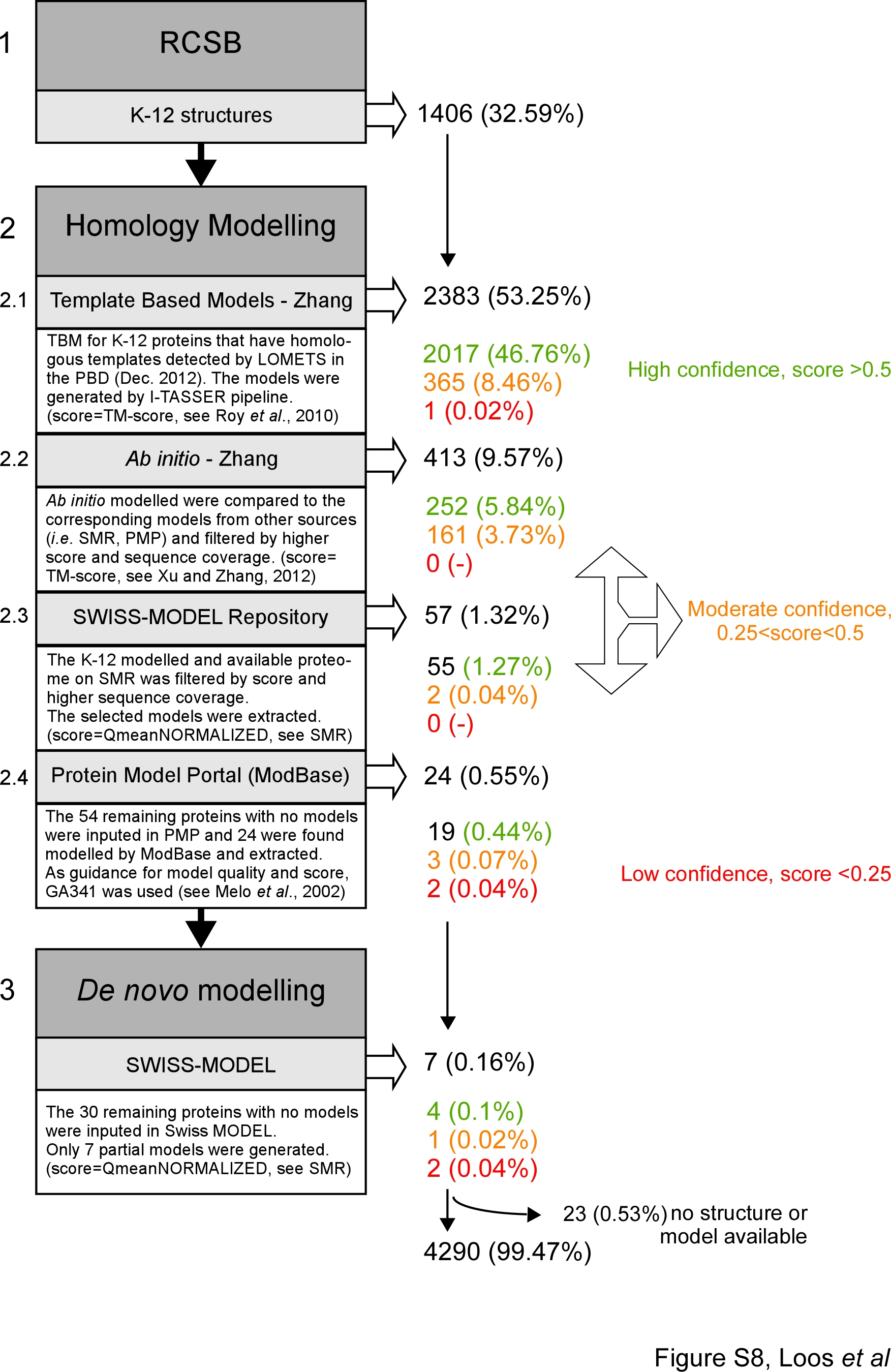


**Figure S8 Workflow *E. coli* K-12 structures and models (related to Table S7 and 8)**

For the listing of all protein structures available for K-12, the Protein Data Bank (RCSB-PBD; (Berman *et al.* 2000; Rose *et al.* 2017)) was filtered for K-12 Uniprot entries (4313). Only 1406 K-12 proteins have experimental structures with ≥30% coverage solved by X-ray, NMR and/or EM (workflow: STEP 1). Homology based models of the remaining 2907 proteins were searched in specialized modelling websites and models repositories, like <https://zhanglab.ccmb.med.umich.edu/Ecoli/>, <https://swissmodel.expasy.org/repository> and <https://www.proteinmodelportal.org/> (workflow: STEP 2). All sorted models possess a quality estimation score (0-1) that have been used to classify them in our list as high confidence models (score≥0.5, colour green), moderate confidence (0.25≤score≤0.5, colour orange) and low confidence (score≤0.25, colour red; Table S8). Zhang proposed TBMs and *ab initio* models (Roy *et al.* 2010; Xu and Zhang 2013). The TBMs generally have homologous templates in the PDB library that can be detected by LOMETS (Wu and Zhang 2007). The continuous modelled fragments are then excised and used to re-assemble the full-length models by the [I-TASSER](http://zhanglab.ccmb.med.umich.edu/I-TASSER/) simulations. Finally, [FG-MD](http://zhanglab.ccmb.med.umich.edu/FG-MD/) simulations are used to refine the atomic models (Roy *et al.* 2010). On the other hand, the *ab initio* models have no homologous templates that can be detected by LOMETS from the PDB library, and they are judged as hard distant-homology proteins. The structural models were generated by QUARK based structure assembly simulations (Xu and Zhang 2013). Both 3784 TBMs and 495 *ab initio* models were screened and sort according to their TM-score (workflow: STEP 2.1 and 2.2). This search in Zhang’s proposed models yielded 2383 K-12 modelled proteins in total. 81 proteins have homology based models from other repository site, like SWISS-MODEL (SMR) and Protein Model Portal (PMP). The SWISS-MODEL repository (Bienert *et al.* 2017) gives access to 6376 K-12 models, which need to be filtered for unique models. Among these 81 models, 57 are extracted from SMR (workflow: STEP 2.3). We could classify 55 (1.27%) models having a high confidence score and only 2 (0.04%) showing moderate confidence. On the other hand, from Protein Model Portal (PMP) 24 more models were derived. Those models generally show a sufficiently good confidence score, apart for 2 models (workflow: STEP 2.4). Those 24 models were generated using ModBase (Pieper *et al.* 2014), an automated modelling pipeline relying on the programs PSI-BLAST (Altschul *et al.* 1997) and MODELLER (Webb and Sali 2016). 19 (0.44%) out of 24 models are classified having high confidence, 3 (0.07%) having moderate confidence and 2 (0.04%) having low confidence. *De novo* modelling using SWISS-MODEL (Biasini *et al.* 2014) was performed on 30 K-12 sequences that had no models or structures available (workflow: STEP 3). Excluding 23 cases of failed modelling due to absence of homologue targets of sufficient quality and proteins being shorter than 30 residues, we were able to generate 7 (0.16%) partial new models, of which 4 (0.1%) with high confidence, 1 (0.02%) with moderate confidence, 2 (0.04%) with low confidence but mostly showing incomplete sequence coverage. The total number of K-12 proteins with structures or models sums up to 4290, corresponding to 99.47% of the full proteome. Abbreviations: PMP = Protein Model Portal; SMR = SWISS-MODEL Repository; TBM = Template based model.

# 2.2 Supplementary Tables

**Table S18 Features that differentiate cytoplasmome from secretome selected by machine learning**

Protein features selected by the JAD Bio machine learning tool (Tsamardinos *et al.* 2018) in the order of importance and the reference where these data can be found in the Supplemental tables.

| **Variable contribution order** | **Protein features selected for topology classification** | **Tool** | **Primary dataset;Reference** |
| --- | --- | --- | --- |
| 1 | Disorder prediction score (normalized by length) | IUPred2 (Meszaros *et al.* 2018) | Table S3 |
| 2 | Met (%) |  | Table S3 |
| 3 | Glu (%) |  | Table S3 |
| 4 | Arg (%) |  | Table S3 |
| 5 | His (%) |  | Table S3 |
| 6 | Tyr (%) |  | Table S3 |
| 7 | Asn (%) |  | Table S3 |
| 8 | Gln (%) |  | Table S3 |
| 9 | Ser (%) |  | Table S3 |
| 10 | Ile (%) |  | Table S3 |
| 11 | Average prediction score of early foldons | EFoldMine (Raimondi *et al.* 2017) | Table S9 |
| 12 | Average energetic cost per residue (normalized by length) |  | Table S3; (Li *et al.* 2009) |
| 13 | mRNA half-life (min) |  | Table S3; (Esquerre *et al.* 2015) |
| 14 | β folds per protein | SUPERFAMILY (Wilson *et al.* 2009) | Table S3 |
| 15 | Leu (%) |  | Table S3 |
| 16 | Thr (%) |  | Table S3 |
| 17 | Asp (%) |  | Table S3 |
| 18 | Essential proteins |  | Table S3; (Goodall *et al.* 2018) |
| 19 | Average hydrophobicity score of hydrophobic patches | GRAVY (Kyte and Doolittle 1982) | Table S9 |
| 20 | α helix content (%) |  | Table S3 |
| 21 | Disorder group (IDP, IDR or no IDRs) | IUPred2 (Meszaros *et al.* 2018) | Table S9 |
| 22 | Protein sequence coverage by IDRs (%) | IUPred2 (Meszaros *et al.* 2018) | Table S9 |
| 23 | α/β folds per protein | SUPERFAMILY (Wilson *et al.* 2009) | Table S3 |
| 24 | Cys (%) |  | Table S3 |

# 3 Supplementary Materials and Methods and Results

**Additional bacterial strains**

Other strains are implemented in STEPdb2.0. BL21(DE3) (Uniprot Proteome ID UP000002032, date 27/2/2018; (Makino *et al.* 2011)) and enteropathogenic *E. coli* (EPEC), strain E2348/69 (Uniprot Proteome ID UP000008205, date 14/4/2017).

**Universal protein names**

Protein names and topologies in Uniprot do not always follow a systematic nomenclature (*e.g.* peripheral IM proteins can be labelled as “Cell inner membrane”, “Cell membrane” and “Membrane”; Table S3). STEPdb 2.0 introduces a uniform naming scheme (similar to that for the *Streptomyces* SToPSdb (Tsolis *et al.* 2018)) which includes subcellular topological information in protein names (Table S3), following the general formalism: organelle, “subcellular topology”, export system (if secreted), function (if known), structural features (if known) and protein name. For example, “cytoplasmic protease YegQ” and “OM lipoprotein & extra-cellular peripheral OM protein (SEC through IM, inserted in OM by LOL), Lpp”.

**Cataloguing a list of the proteostatic machinery: chaperones and folding factors**

The proteostasis network comprises chaperone systems that can cooperate, compensate for each other, overlap in client proteins and have often more than one function (Dickson and Brooks 2013). Some chaperones will only be activated in cases of specific stress, *e.g.* acid (HdeA and HdeB (Malki *et al.* 2008)), secretion stress (Spy (Quan *et al.* 2011)) or oxidative stress (Hsp33 (Jakob *et al.* 1999)) and others will even act as disaggregases to untangle denatured polypeptides (Doyle *et al.* 2013). Occasionally, chaperones fail in their roles, *e.g.* because a client polypeptide has been over-synthesized (Cho *et al.* 2015) or is damaged by covalent modifications (Ross and Poirier 2004) or because the external stress has been too excessive (Cho *et al.* 2015). In those cases, polypeptides may misfold and aggregate forming structures like inclusion bodies or amyloid fibrils (Kopito 2000; Baneyx and Mujacic 2004). Misfolded and aggregated proteins are brought back to their functional, natively folded state via collaborating disaggregases, holdases and foldases (Cho *et al.* 2015).

A class of periplasmic folding factor has a dedicated role in cell envelope folding. cell envelope folding is challenging due to the crowded, oxidizing environment, varying pH and lack of ATP. Different proteins in the cell envelope undergo different folding pathways assisted by a certain set of chaperones and folding factors. Lipoproteins use the LOL (localization of lipoproteins) system comprising the chaperones LolA, LolB next to LolCDE (Okuda and Tokuda 2011). Soluble periplasmic proteins are assisted in their folding by dedicated chaperones and folding catalysts like PPIases and disulfide-bond-forming enzymes. OM proteins rely on chaperones to traverse the periplasm in a soluble unfolded form but often only reach their final folded conformation in the hydrophobic milieu of the membrane.

In K-12, 124 proteins constitute the chaperonome, distributed in all cellular compartments including membranes (Table S5). Of these, 92 are protein chaperones. 15 chaperone RNA and 11 metals (copper, manganese, zinc and iron-sulfur protein biogenesis). Many chaperones are found at elevated concentrations that match those of the most abundant proteins in K-12 and equal or exceed those of ribosomes even under normal growth conditions (Table S5; (Schmidt *et al.* 2016; Tsirigotaki *et al.* 2017)), with GroL, GroS and DnaK being the most abundant chaperones (Santra *et al.* 2017). Others are part of cellular stress responses and may elevate their concentrations several folds upon shock (Arsene *et al.* 2000; Guisbert *et al.* 2004; Mujacic and Baneyx 2006; Luders *et al.* 2009; Richter *et al.* 2010).

In *E. coli* 6 folding networks are at play (Baneyx and Mujacic 2004; Meyer and Baker 2011; Calloni *et al.* 2012; Cho *et al.* 2015). Native (*de novo*) folding in the cytoplasm is being taken care of by DnaK (1), GroEL/ES (2) and TF (3). The DnaK/DnaJ/GrpE system also handles misfolded proteins and takes them back to an unfolded state. DnaK/DnaJ/GrpE is assisted by the disaggregase ClpB (4) and, in stress situations, also by the holdases Hsp31, Hsp33 and IbpA/B (5). These proteostasis networks are dynamic, intertwined and change as a function of protein translation (more clients) and stressors (upregulated expression of chaperones/proteases) as illustrated by computational modelling (Powers *et al.* 2012). Secretory proteins, often translocated to the periplasm and beyond in unfolded conformations, reach their final natively folded conformation with the help of the cell envelope proteostasis network (6) consisting of Skp, FkpA, SurA and the Dsb system (Baneyx and Mujacic 2004).

**The proteostatic machinery: proteases**

83 proteases and peptidases in K-12 operate together with and next to chaperones to maintain proteostasis both under physiological and stress conditions (Table S6; (Tomoyasu *et al.* 2001; Meyer and Baker 2011)). Some (12) have a specific substrate (*e.g.* RseP, Prc) but most work on a broad range of substrates often with a certain sequence specificity. 14 proteases are in involved in cell wall biogenesis/remodelling/recycling. A few proteases/peptidases are dedicated to one specific task: *e.g.* Map removes the N-terminal methionine residue from nascent chains and LepB, LspA and GspO cleave secretory protein signal peptides.

A network of proteases and peptidases involved in the normal protein quality control (PepN, HslV, FtsH, BepA, ClpP and - the most important one - Lon) degrades misfolded and aged proteins back to amino acids that will re-enter biosynthetic circulation on ribosomes. Under stress conditions this network becomes even more important and is assisted additionally by DegP and DegQ.

**Protein characteristics analysis**

Backbone dynamics was predicted as described previously with DynaMine tool (Cilia *et al.* 2013).

EFoldMine tool was used to predict early folding propensity as described previously (Raimondi *et al.* 2017). The scores for individual amino acids were classified in two groups: amino acids with scores >0.169 were assigned as early folding residues, while the ones with scores ≤0.169 were not.

Intrinsic disorder per residue of the protein sequence was obtained from IUPred2 (Meszaros *et al.* 2018) and hydrophobicity scale of each residue of the protein is assigned from Kyte-Doolittle scale (GRAVY tool; (Kyte and Doolittle 1982)). To predict the IDRs and hydrophobic segments a minimum window of 5 residues was set and a sliding window was used to calculate the scores.

Foldons, IDRs, hydrophobic segments were predicted with sliding window. The average of Early folding prediction/IUPred2/K&D scores of each residue is calculated for the window and assigned to the middle residue for respective region. The algorithm then moves down per residue calculate the average, assign it to the middle of the sliding window and this process repeats down the sequence. The first and last two residues were left out in the process. Therefore, the first two residues were assigned an average of their Kyte-Doolitte/IUPred2 scores; the same is applied for last two residues in a protein sequence. Consecutive residues with scores above 0 were considered as hydrophobic segments. Consecutive residues with scores above 0.5 were considered as IDRs. Segments of size at least 5 were only considered for analysis.

For the aggregation propensities, Aggregation prone regions (APRs) and gatekeeper residues we used TANGO algorithm (Fernandez-Escamilla *et al.* 2004). Gatekeeper residues were defines as the N-terminal and C-terminal residues neighbouring the APRs (Beerten *et al.* 2012). Average frequency of amino acid residues was calculated in gatekeeper regions without discriminating between N-terminal and C-terminal ones. Here, we recalculated the TANGO score for APR regions in order to correct any influence from the properties of the flanking gatekeeper residues and is defined as pure TANGO score. To calculate pure TANGO, the gatekeeper residues were replaced by Alanine. The resultant is APR region flanked by alanine on both side. The Tango scores of these segments normalized by the length of the segment are called pure Tango.

Other characteristics were added obtained from different studies. Protein melting temperatures (Tm) were obtained from limited proteolysis and mass spectrometry analyses (Leuenberger *et al.* 2017). Inherent solubility was obtained from the eSOL database (Niwa *et al.* 2009). Protein expression data were integrated from the mass-spectrometry-based integrated *E. coli* dataset (Schmidt *et al.* 2016).

Other factors that influence secretome polypeptide folding at final destinations (Tsirigotaki *et al.* 2018) are multiple factors including disulphide bonds, ligands and oligomerization (Kadokura and Beckwith 2010). We have included this information in Table S3 that we obtained from literature of our own analysis (see below for disulphide bonds).

**Comparison of disorder prediction by IUPred2 and MobiDB tools to experimentally annotated proteins in Disprot**

In order to test the performance of a disorder predictor tool for proteome-wide analysis we compared MobiDB (Piovesan *et al.* 2018) and IUPred2 (Meszaros *et al.* 2018) against the experimentally determined disordered proteins from Disprot (Piovesan *et al.* 2017). Therefore we have compared the coverage by IDRs as reported on the Disprot database to the percentage of amino acids that are predicted to be disordered by IUPred2 and MobiDB (Table S9, columns S, T and U, respectively). We have observed that the proteins with high coverage by IDRs as annotated by Disprot are also predicted to have more disordered amino acids by IUPred2 and MobiDB. On the other hand, Disprot annotated proteins with less disordered regions are also predicted to have less disordered amino acids by both IUPred2 and MobiDB. Therefore we concluded that both prediction tools agree and as shown in Figure S3 reveal the same trends. We decided to use IUPred2 for further analysis (see main text for more details).

**Cataloguing a comprehensive list of the K-12 structural proteome**

For the listing of all protein structures available for K-12, the Protein Data Bank (RCSB-PBD; (Berman *et al.* 2000; Rose *et al.* 2017)) was filtered for unique K-12 Uniprot entries. To date, the Protein Data Bank only 1406 high resolution structures (32.59% of the proteome) have a ≥30% coverage (Fig. S8; 106 have 30-70% coverage calculated after removal of signal peptides; Table S7 and S8).

Homology based models of the remaining proteins were searched in specialized modelling websites, repositories, like (<https://zhanglab.ccmb.med.umich.edu/Ecoli/>), (<https://swissmodel.expasy.org/repository>) and (<https://www.proteinmodelportal.org/>).

Zhang proposed Template Based Models (TBMs) and *ab initio* models (Roy *et al.* 2010; Xu and Zhang 2013). The TBMs generally have homologous templates in the PDB library that can be detected by LOMETS (Wu and Zhang 2007), while the *ab initio* models have none. For the TBM models, the continuous modelled fragments are excised and used to re-assemble the full-length models by the [I-TASSER](http://zhanglab.ccmb.med.umich.edu/I-TASSER/) simulations. [FG-MD](http://zhanglab.ccmb.med.umich.edu/FG-MD/) simulations are used to refine the atomic models (Roy *et al.* 2010). On the other hand, the *ab initio* models were generated by QUARK based structure assembly simulations (Xu and Zhang 2013). Both TBMs (3784) and *ab initio* models (495) were screened and sort according to their TM-score. TM-score measures the global fold similarity and is less sensitive to the local structural variations, varying between 0 and 1, where 1 indicates a perfect match between the model and the target protein. TM-score ≥0.5 assumes generally the same fold, TM-score ≥0.35 suggests a substantial portion of structure correctly modelled whereas TM-score ≤0.17 corresponds to randomly chosen unrelated proteins. This search in Zhang’s proposed models yielded 2739 K-12 modelled proteins in total because of two reasons: 1459 are now available in the RCSB-PBD and therefore have been listed as hard data; 81 have been excluded because of the low modelling score with I-TASSER but good enough modelling score and sequence coverage using SWISS-MODEL. Another 413 (9.57%) models derived *ab initio, i.e.* without any structural template (Xu and Zhang 2013), were classified as “hypothetical” until further validation.

For 51 RCSB-PDB structures with coverage <30% (*e.g.* P29131) we chose available models over the incomplete PDB structure after manual examination.

For the remaining proteins with unavailable structures, models were derived based on “threading” to existing homologous structure templates (Table S7 and S8; (Brunk *et al.* 2016)) or on residue co-evolution (Kamisetty *et al.* 2013) or *ab intitio,* template-independent (see Supplemental Experimental Procedures). Collectively, these efforts yielded structural models (of >30% coverage) for 2884 K-12 proteins (99.47% of the proteome). Models were classified according to confidence statistics as high (green), middle (orange) and low (red) (Fig. S8; Table S7 and S8).

The SWISS-MODEL repository (Bienert *et al.* 2017) gave access to 6,376 K-12 models, which were filtered for unique models. As SWR evaluation criteria we used the normalized QMEAN quality score (Benkert *et al.* 2008). The same is valid for Protein Model Portal (PMP) from which other 22 models were derived of sufficiently good confidence score, apart for 2 models. Those models were generated using ModBase (Pieper *et al.* 2014), an automated modelling pipeline relying on the programs PSI-BLAST (Altschul *et al.* 1997) and MODELLER (Webb and Sali 2016). Special care needs to be taken to assess the quality of those models, because more than one models is provided per sequence. This can happen if the sequence got processed in different datasets (at different times, for a different project, *etc.*) or if there are models for different domains. The criteria applied for selecting these models were: 1. good sequence identity to the target; 2. higher sequence coverage; 3. higher quality estimator score. In fact, more than one quality estimator score is given as output. Here, a reliability score called GA341 (Melo *et al.* 2002) has been chosen as one criteria. If GA341≥0.7, a reliable model has a probability of the correct fold that is larger than 95%. A fold is correct when at least 30% of its Cα atoms superpose within 3.5 Å of their correct positions (Pieper *et al.* 2014).

*De novo* modelling using SWISS-MODEL (Biasini *et al.* 2014) was performed on the remaining K-12 sequences that had no models or structures available. Excluding 23 cases of failed modelling due to absence of homologue targets of sufficient quality and proteins being shorter than 30 residues, we were able to generate 7 partial new models of reasonable quality.

A single PDB ID of the best scoring models (Reference structure) is provided in STEPdb 2.0; links to more top-scored models on the Zhang lab WWW site are provided online. 4 *ab initio* models were revised: Two of these models (E10369 and E13645 corresponding to Uniprot codes P77551 and P33345) presented a fused sequence of two different proteins, one (E10645; Uniprot code P0ADQ7) had 4 extra N-terminal amino acids and one (E10558) was discarded because it did not correspond to an *E.coli* sequence (Uniprot code P76164).

Upon searching the Swiss Model (Bienert *et al.* 2017) and Protein Model Portal (Haas *et al.* 2013) repositories, we obtained another 81 unique models (Fig. S8, Table S7) that had robust evaluation criteria (QMEAN and GA341 score; see Supplemental Experimental Procedures). We therefore could classify 55 SWR models (1.27%) having a high confidence score and only 2 (0.04%) having moderate confidence. Among the remaining 24 PMP-PSI KB ModBase models (0.55%), 19 models (0.44%) were classified with high confidence, 3 (0.07%) with moderate confidence and 2 (0.04%) with low confidence.

23 proteins, 13 of which are involved in stress response (Hemm *et al.* 2010), remain with no possible structure prediction (more information about these proteins is online: http://stepdb.eu/). These include several small IM spanning or peripherally associated proteins (Hemm *et al.* 2008; Fontaine *et al.* 2011).

**Structural data analysis**

For the structural analysis, we included PDBx/mmcif format (Berman *et al.* 2000). PDBe SIFT service is used to get the mapping between the structures and Uniprot sequences (Dimmer *et al.* 2012).

Absolute and relative contact orders of each protein, describing the compactness of the folded structure (Shi *et al.* 2008), was calculated as described (Plaxco *et al.* 1998). Relative CO was calculated from PDB structures by determining average sequence distances between amino acids that form native contacts, divided by protein length (within 6 Å for each amino acid). Only experimentally solved structures (from RCSB-PDB) were used having coverage ≥30%.

Small fast folding domains may initiate folding in the ribosome exit tunnel (Marino *et al.* 2016), while large proteins will have to be either first fully synthesized before the major folding steps initiate or folding may proceed while the nascent chain exits the ribosome (Kudva *et al.* 2018). For the Sec-dependent secretome, folding is *de facto* post-translational and will only occur once the chains leave the exit mouth of the SecY channel, with the C-terminus last (Tsirigotaki *et al.* 2017). In all cases, the C-termini will play important part in finalizing folding, particularly for single domain proteins. C-termini, providing important contribution to the fold, come in close proximity to N-termini (Fig. S6: *e.g.* cytoplasmic TusB in panel B; periplasmic PpiA in panel C; Table S3). Distances between N- and C-termini for the 235 1-fold proteins with solved structures of 100% coverage (Table S8) are on average 2.7 nm. Secreted proteins without OM proteins have slightly longer N to C termini separation (Fig. S6A), but the difference is not significant. For proteins with close N- to C- distances, folding cannot be completed before complete synthesis, or full release from SecY. The same is true for all single domain OM β-barrels that require both OM embedding and for their first/last β-strands to H-bond. OM protein distances are much shorter compared to other classes (Fig. S6A).

**The Disulfideome**

Disulfide bonds mostly form outside the reducing environment of the cytoplasm (Hogg 2003; Kadokura *et al.* 2003) and are controlled by thiol-disulfide oxidoreductases (TDORs). These enzymes are not essential for growth but their loss causes various phenotypic defects (Kadokura *et al.* 2003). In *E. coli* cytoplasmic disulfides are dealt with by the thioredoxin and the glutathione/glutaredoxin pathways (Prinz *et al.* 1997). Disulfide formation in the cell envelope involves the DsbA-DsbB (generating) and the DsbC-DsbD (repairing) systems (Kadokura and Beckwith 2010). Periplasmic DsbA generates a disulfide bond in the secreted client as or after it crosses the IM. Oxidase DsbA has a catalytic CXXC motif in a thioredoxin-fold (Martin 1995). Once the disulfide bond is formed in the substrate, DsbA is left in the reduced state. DsbB, a redox partner of DsbA, will regenerate it to the active state. DsbB itself will recover by transferring electrons to membrane-residing ubiquinone (Lee and Davey 2017). When there are more than two cysteines in a protein non-native bonds can be formed. These are reduced and corrected by DsbC. DsbD is the redox partner of DsbC (Kadokura and Beckwith 2010). Without these two pathways bacterial proteins undergo slower or no disulfide formation, or form uncorrected wrong bonds (Kadokura and Beckwith 2010).

Although cytoplasmic proteins have significantly more cysteines, secreted proteins have a bias towards carrying even numbers of cysteines as observed previously (Table S3; (Dutton *et al.* 2008)).

K-12 has 242 periplasmic proteins containing ≥2 cysteines. High-throughput mass spectrometry identified 199 disulfide bonds in 150 periplasmic proteins in strain BW25113 (Table S3; (Lu *et al.* 2015)). Of these, 148 are have homologous proteins in MG1655 strain. Deletion analysis revealed 72 and 15 substrates as DsbA and DsbC substrates, respectively.

Potential disulfide bonds (distance ≤3 Å) and corresponding torsions for each (Cβ1-S1-S2-Cβ2) were calculated from PDB structures. We have analyzed the proximity of cysteine residues in experimentally solved structures and identified that 84 pairs of cysteines are located within 3 Å from each other. Secreted proteins have shown a clear distribution of torsion angles between the first C-atoms, usually being around 80 to 120 degrees. 33 out of 84 proteins we found to have potential SS bonds are cytoplasmic (1.2% of cytoplasmome). After manual checking of 10 structures with identified SS bond, we verify that such SS bonds are indeed possible. Finally, we observed that the torsion angles in cytoplasmome are not as defined as in the secretome (Table S3).

**Change in protein abundance**

Change in protein abundance using variability score was calculated using protein concentration data derived from growth conditions described by Schmidt *et al* (Schmidt *et al.* 2016). We have focussed on 13 growth conditions only that include growth on minimal media with excess of different carbon and energy sources, LB and a complex medium. These conditions are: minimal media with excess of acetate, fumarate, galactose, glucose, glucosamine, glycerol, pyruvate, succinate, fructose, mannose or xylose, LB medium or glycerol + amino acid complex medium as described in Schmidt *et al*. No stress conditions were used in order to keep the different datasets comparable. Variability score was calculated based on coefficient of variation and pairwise fold change across all conditions.

Pairwise fold change: Pairwise fold-change is checked for 13 different conditions. The number of times the expression goes beyond 2.5-fold is then counted and normalized by the total number of pairwise conditions considered. This value is then added to the CV and is normalized in the 0 to 1 scale, called variability score (VS) and defined the following change in protein abundance classes: very low (VS≤0.02), low (VS≤0.05), moderate (0.05<VS≤0.4) and high (VS>0.4).

Gene Ontology and functional analysis were performed by PANTHER (Wilson *et al.* 2009). For that, protein lists from different abundance classes were used as input in PANTHER. The hits were obtained for 3 different criteria: Molecular function, Biological process and Protein class. This was downloaded from the server as protein lists and we calculated percentage of found groups.

**Treatment of ribosome profiling data**

Ribosome profiling method is limited by translated substrate and interacting factor abundance and isolated complex stability and purity, that can lead to false positive interactions and are challenging to assess (Becker *et al.* 2013).

For the identification of the SRP interactome, *Schibich et al.,* (Schibich *et al.* 2016) used two approaches with distinct thresholds: 1) the ratio-based enrichment of RNCs with a threshold of 2fold (SRP-RNCs/cellular RNCs≥2), and 2) the peak detection that monitors the enrichment ratio over a window of 11 nucleotides, with a threshold of 5 for reproducibly detected peaks in two replicates (Pearson correlation >0.6). RNCs passing either of the two thresholds were considered by *Schibich et al.,* as SRP-substrates, proposing that 566 proteins specifically interact with SRP co-translationally, among the 2367 detected nascent proteins in the cellular translatome. SRP-interactors comprised primarily of integral membrane proteins, as well as 14 secretory proteins 50 cytoplasmic proteins and 14 of unknown subcellular localization (Schibich *et al.* 2016).

For the integration of the SRP-substrates into STEPdb 2.0, the list of the detected genes reported by *Schibich et al*. was matched to primary gene names from the NCBI reference genome sequence NC_012759.1 (version 2013) (*E. coli* BW2952), and was subsequently matched to the *E. coli* K12 MG1655 genome (provided by the Uniprot database) using primary or alternative gene names. Manual curation using the Blast function of the Expasy server (<https://web.expasy.org/blast/>) was required for a few genes for confident matching (*e.g*. gene *dgsA* in *E. coli* BW2952 was matched to the *mlc* gene in *E. coli* K-12 MG1655). SRP recognition profiles were further filtered for higher confidence, using a dual pass approach, due to the limitations of ribosome profiling and the current absence of robust statistical analysis. Specifically, only genes that were identified as SRP-substrates by both detection analysis by *Schibich et al.,* are considered in our dual pass approach as SRP-substrates (Table S16). Similarly, genes that were considered by *Schibich et al.,* as not SRP-interactors but had low reproducibility in the two experimental replicates (Pearson correlation <0.6) are omitted from the dataset (noted as “NO” in column I of Table S16). The dual pass approach identifies 379 SRP-interactors (Fig. 7B-C; Table S16).

A similar approach was applied for matching of the TF-interactome, to the *E. coli* K12 MG1655 proteome. The same criteria as those reported by *Oh et al.,* (Oh *et al.* 2011) were applied for the annotation of the TF-interactome, that is a 1.3 fold enrichment ratio threshold (TF-RNCs/cellular RNCs≥1.3), as the enrichment factor was the only analysis method used by *Oh et al.* For the co-translational SecA-interactome, the identified genes by *Huber et al.,* (Huber *et al.* 2017) were matched to the *E. coli* K-12 MG1655 genome using gene accession numbers, and interactors were annotated similarly to the process followed for the TF-interactome. In all cases, subcellular protein localization refers to the STEPdb annotation, unless a references is specifically cited (*e.g.* original, unfiltered conclusions of the SRP-interactome reported by *Schibich et al.,* (Schibich *et al.* 2016)). Despite their very low fractional abundance, OM proteins were proposed to be strong TF- interactors based on their enrichment factors (*e.g.* LptD, OmpF, LamB enrichment factor: 2.5; average enrichment factor of total TF-interactome: 2.4; threshold: 1.3), and on the impairment of OM integrity in Δ*tig* cells (Oh *et al.* 2011).

A similar approach with additional step of cross-linking identified 1,305 nascent SecA interactors (Huber *et al.* 2017). These nascent proteins were enriched >1.3 fold (SecA-RNCs/cellular RNCs ≥ 1.3) taken as indicative of co-translational interaction with SecA (Table S16).

**Cataloguing soluble chaperone interactors**

The interactors of soluble chaperones TF, DnaK and GroEL were obtained from the literature (Supplemental Experimental Procedures; (Butland *et al.* 2005; Kerner *et al.* 2005; Arifuzzaman *et al.* 2006; Chapman *et al.* 2006; Martinez-Hackert and Hendrickson 2009; Calloni *et al.* 2012; Niwa *et al.* 2012)).

Determining the interactors of three main soluble chaperones (TF, DnaK, GroEL) was based on primarily by pull down proteomics approaches followed by mass spectrometry (Fig. S7C-E; (Arifuzzaman *et al.* 2006; Martinez-Hackert and Hendrickson 2009; Hartl *et al.* 2011; Calloni *et al.* 2012)) and the determination of soluble/aggregated proteomes after removal of chaperone genes (Deuerling *et al.* 1999; Martinez-Hackert and Hendrickson 2009; Niwa *et al.* 2012). A third approach studied large scale chaperone-interactions in *in vitro* reconstituted systems (Fig. 7E; (Calloni *et al.* 2012; Niwa *et al.* 2012)).

For some cytoplasmic residents, TF may act as a holdase to prevent co-translational misfolding until it passes them on to foldases TF, DnaK and GroEL “interactors” specificities have been determined experimentally by multiple methods, including proteomics, yielding convoluted results (Table S17; Supplemental Experimental Procedures). Even for the same chaperone, different studies, fail to return the same interactors (Fig. S7F). Some chaperones only have a few interactors, while other interactors interact mainly with one or with multiple chaperones (Mapa *et al.* 2012).

Chaperones may have primarily one (DnaK) or multiple (GroEL, Trigger factor) client binding sites (CBS). These are short peptide segments, commonly hydrophobic (Perrett *et al.* 1997; Koldewey *et al.* 2017) but not always (Saio *et al.* 2014), recognized on these CBS with one (Randall *et al.* 1998; Huang *et al.* 2016) or multiple (Calloni *et al.* 2012; Saio *et al.* 2014) chaperones binding on the same client chain forming freely dissociable complexes. GroEL forms a closed chamber, for proteins <70 kDa (Chaudhuri *et al.* 2009).

**Statistical analysis**

For discrete variables, we used Chi-square and Fisher’s exact tests; for continuous variables we used Kruskal Wallis and Wilcoxon tests. Bonferroni correction was used to adjust the p-value for the multiple testing hypothesis.

**4 References**

Altschul, S. F., T. L. Madden, A. A. Schaffer, J. Zhang, Z. Zhang, W. Miller and D. J. Lipman (1997). "Gapped BLAST and PSI-BLAST: a new generation of protein database search programs." Nucleic Acids Res 25(17): 3389-3402.

Arifuzzaman, M., M. Maeda, A. Itoh, K. Nishikata, C. Takita, R. Saito, T. Ara, K. Nakahigashi, H. C. Huang, A. Hirai, K. Tsuzuki, S. Nakamura, M. Altaf-Ul-Amin, T. Oshima, T. Baba, N. Yamamoto, T. Kawamura, T. Ioka-Nakamichi, M. Kitagawa, M. Tomita, S. Kanaya, C. Wada and H. Mori (2006). "Large-scale identification of protein-protein interaction of Escherichia coli K-12." Genome Res 16(5): 686-691 doi: 10.1101/gr.4527806.

Arsene, F., T. Tomoyasu and B. Bukau (2000). "The heat shock response of Escherichia coli." Int J Food Microbiol 55(1-3): 3-9.

Baneyx, F. and M. Mujacic (2004). "Recombinant protein folding and misfolding in Escherichia coli." Nat Biotechnol 22(11): 1399-1408 doi: 10.1038/nbt1029.

Becker, A. H., E. Oh, J. S. Weissman, G. Kramer and B. Bukau (2013). "Selective ribosome profiling as a tool for studying the interaction of chaperones and targeting factors with nascent polypeptide chains and ribosomes." Nat Protoc 8(11): 2212-2239 doi: 10.1038/nprot.2013.133.

Beerten, J., J. Schymkowitz and F. Rousseau (2012). "Aggregation prone regions and gatekeeping residues in protein sequences." Curr Top Med Chem 12(22): 2470-2478.

Benkert, P., S. C. Tosatto and D. Schomburg (2008). "QMEAN: A comprehensive scoring function for model quality assessment." Proteins 71(1): 261-277 doi: 10.1002/prot.21715.

Berman, H. M., J. Westbrook, Z. Feng, G. Gilliland, T. N. Bhat, H. Weissig, I. N. Shindyalov and P. E. Bourne (2000). "The Protein Data Bank." Nucleic Acids Res 28(1): 235-242.

Biasini, M., S. Bienert, A. Waterhouse, K. Arnold, G. Studer, T. Schmidt, F. Kiefer, T. Gallo Cassarino, M. Bertoni, L. Bordoli and T. Schwede (2014). "SWISS-MODEL: modelling protein tertiary and quaternary structure using evolutionary information." Nucleic Acids Res 42(Web Server issue): W252-258 doi: 10.1093/nar/gku340.

Bienert, S., A. Waterhouse, T. A. de Beer, G. Tauriello, G. Studer, L. Bordoli and T. Schwede (2017). "The SWISS-MODEL Repository-new features and functionality." Nucleic Acids Res 45(D1): D313-D319 doi: 10.1093/nar/gkw1132.

Brunk, E., N. Mih, J. Monk, Z. Zhang, E. J. O'Brien, S. E. Bliven, K. Chen, R. L. Chang, P. E. Bourne and B. O. Palsson (2016). "Systems biology of the structural proteome." BMC Syst Biol 10: 26 doi: 10.1186/s12918-016-0271-6.

Butland, G., J. M. Peregrin-Alvarez, J. Li, W. Yang, X. Yang, V. Canadien, A. Starostine, D. Richards, B. Beattie, N. Krogan, M. Davey, J. Parkinson, J. Greenblatt and A. Emili (2005). "Interaction network containing conserved and essential protein complexes in Escherichia coli." Nature 433(7025): 531-537 doi: nature03239 [pii] 10.1038/nature03239.

Calloni, G., T. Chen, S. M. Schermann, H. C. Chang, P. Genevaux, F. Agostini, G. G. Tartaglia, M. Hayer-Hartl and F. U. Hartl (2012). "DnaK functions as a central hub in the E. coli chaperone network." Cell Rep 1(3): 251-264 doi: 10.1016/j.celrep.2011.12.007.

Chapman, E., G. W. Farr, R. Usaite, K. Furtak, W. A. Fenton, T. K. Chaudhuri, E. R. Hondorp, R. G. Matthews, S. G. Wolf, J. R. Yates, M. Pypaert and A. L. Horwich (2006). "Global aggregation of newly translated proteins in an Escherichia coli strain deficient of the chaperonin GroEL." Proc Natl Acad Sci U S A 103(43): 15800-15805 doi: 10.1073/pnas.0607534103.

Chaudhuri, T. K., V. K. Verma and A. Maheshwari (2009). "GroEL assisted folding of large polypeptide substrates in Escherichia coli: Present scenario and assignments for the future." Prog Biophys Mol Biol 99(1): 42-50 doi: 10.1016/j.pbiomolbio.2008.10.007.

Cho, Y., X. Zhang, K. F. Pobre, Y. Liu, D. L. Powers, J. W. Kelly, L. M. Gierasch and E. T. Powers (2015). "Individual and collective contributions of chaperoning and degradation to protein homeostasis in E. coli." Cell Rep 11(2): 321-333 doi: 10.1016/j.celrep.2015.03.018.

Cilia, E., R. Pancsa, P. Tompa, T. Lenaerts and W. F. Vranken (2013). "From protein sequence to dynamics and disorder with DynaMine." Nat Commun 4: 2741 doi: 10.1038/ncomms3741.

Dana, A. and T. Tuller (2014). "Mean of the typical decoding rates: a new translation efficiency index based on the analysis of ribosome profiling data." G3 (Bethesda) 5(1): 73-80 doi: 10.1534/g3.114.015099.

Deuerling, E., A. Schulze-Specking, T. Tomoyasu, A. Mogk and B. Bukau (1999). "Trigger factor and DnaK cooperate in folding of newly synthesized proteins." Nature 400(6745): 693-696 doi: 10.1038/23301.

Dickson, A. and C. L. Brooks, 3rd (2013). "Quantifying chaperone-mediated transitions in the proteostasis network of E. coli." PLoS Comput Biol 9(11): e1003324 doi: 10.1371/journal.pcbi.1003324.

Dimmer, E. C., R. P. Huntley, Y. Alam-Faruque, T. Sawford, C. O'Donovan, M. J. Martin, B. Bely, P. Browne, W. Mun Chan, R. Eberhardt, M. Gardner, K. Laiho, D. Legge, M. Magrane, K. Pichler, D. Poggioli, H. Sehra, A. Auchincloss, K. Axelsen, M. C. Blatter, E. Boutet, S. Braconi-Quintaje, L. Breuza, A. Bridge, E. Coudert, A. Estreicher, L. Famiglietti, S. Ferro-Rojas, M. Feuermann, A. Gos, N. Gruaz-Gumowski, U. Hinz, C. Hulo, J. James, S. Jimenez, F. Jungo, G. Keller, P. Lemercier, D. Lieberherr, P. Masson, M. Moinat, I. Pedruzzi, S. Poux, C. Rivoire, B. Roechert, M. Schneider, A. Stutz, S. Sundaram, M. Tognolli, L. Bougueleret, G. Argoud-Puy, I. Cusin, P. Duek-Roggli, I. Xenarios and R. Apweiler (2012). "The UniProt-GO Annotation database in 2011." Nucleic Acids Res 40(Database issue): D565-570 doi: gkr1048 [pii] 10.1093/nar/gkr1048.

Doyle, S. M., O. Genest and S. Wickner (2013). "Protein rescue from aggregates by powerful molecular chaperone machines." Nat Rev Mol Cell Biol 14(10): 617-629 doi: 10.1038/nrm3660.

Dutton, R. J., D. Boyd, M. Berkmen and J. Beckwith (2008). "Bacterial species exhibit diversity in their mechanisms and capacity for protein disulfide bond formation." Proc Natl Acad Sci U S A 105(33): 11933-11938 doi: 10.1073/pnas.0804621105.

Dworkin, J. (2009). "Cellular polarity in prokaryotic organisms." Cold Spring Harb Perspect Biol 1(6): a003368 doi: 10.1101/cshperspect.a003368.

Errington, J. (2015). "Bacterial morphogenesis and the enigmatic MreB helix." Nat Rev Microbiol 13(4): 241-248 doi: 10.1038/nrmicro3398.

Esquerre, T., A. Moisan, H. Chiapello, L. Arike, R. Vilu, C. Gaspin, M. Cocaign-Bousquet and L. Girbal (2015). "Genome-wide investigation of mRNA lifetime determinants in Escherichia coli cells cultured at different growth rates." BMC Genomics 16: 275 doi: 10.1186/s12864-015-1482-8.

Fernandez-Escamilla, A. M., F. Rousseau, J. Schymkowitz and L. Serrano (2004). "Prediction of sequence-dependent and mutational effects on the aggregation of peptides and proteins." Nat Biotechnol 22(10): 1302-1306 doi: 10.1038/nbt1012.

Fontaine, F., R. T. Fuchs and G. Storz (2011). "Membrane localization of small proteins in Escherichia coli." J Biol Chem 286(37): 32464-32474 doi: 10.1074/jbc.M111.245696.

Glas, M., H. B. van den Berg van Saparoea, S. H. McLaughlin, W. Roseboom, F. Liu, G. M. Koningstein, A. Fish, T. den Blaauwen, A. J. Heck, L. de Jong, W. Bitter, I. J. de Esch and J. Luirink (2015). "The Soluble Periplasmic Domains of Escherichia coli Cell Division Proteins FtsQ/FtsB/FtsL Form a Trimeric Complex with Submicromolar Affinity." J Biol Chem 290(35): 21498-21509 doi: 10.1074/jbc.M115.654756.

Goodall, E. C. A., A. Robinson, I. G. Johnston, S. Jabbari, K. A. Turner, A. F. Cunningham, P. A. Lund, J. A. Cole and I. R. Henderson (2018). "The Essential Genome of Escherichia coli K-12." MBio 9(1) doi: 10.1128/mBio.02096-17.

Guisbert, E., C. Herman, C. Z. Lu and C. A. Gross (2004). "A chaperone network controls the heat shock response in E. coli." Genes Dev 18(22): 2812-2821 doi: 10.1101/gad.1219204.

Haas, J., S. Roth, K. Arnold, F. Kiefer, T. Schmidt, L. Bordoli and T. Schwede (2013). "The Protein Model Portal--a comprehensive resource for protein structure and model information." Database (Oxford) 2013: bat031 doi: 10.1093/database/bat031.

Hale, C. A. and P. A. de Boer (1999). "Recruitment of ZipA to the septal ring of Escherichia coli is dependent on FtsZ and independent of FtsA." J Bacteriol 181(1): 167-176.

Hartl, F. U., A. Bracher and M. Hayer-Hartl (2011). "Molecular chaperones in protein folding and proteostasis." Nature 475(7356): 324-332 doi: 10.1038/nature10317.

Hemm, M. R., B. J. Paul, J. Miranda-Rios, A. Zhang, N. Soltanzad and G. Storz (2010). "Small stress response proteins in Escherichia coli: proteins missed by classical proteomic studies." J Bacteriol 192(1): 46-58 doi: 10.1128/JB.00872-09.

Hemm, M. R., B. J. Paul, T. D. Schneider, G. Storz and K. E. Rudd (2008). "Small membrane proteins found by comparative genomics and ribosome binding site models." Mol Microbiol 70(6): 1487-1501 doi: 10.1111/j.1365-2958.2008.06495.x.

Hogg, P. J. (2003). "Disulfide bonds as switches for protein function." Trends Biochem Sci 28(4): 210-214 doi: 10.1016/S0968-0004(03)00057-4.

Huang, C., P. Rossi, T. Saio and C. G. Kalodimos (2016). "Structural basis for the antifolding activity of a molecular chaperone." Nature 537(7619): 202-206 doi: 10.1038/nature18965.

Huber, D., M. Jamshad, R. Hanmer, D. Schibich, K. Doring, I. Marcomini, G. Kramer and B. Bukau (2017). "SecA Cotranslationally Interacts with Nascent Substrate Proteins In Vivo." J Bacteriol 199(2) doi: 10.1128/JB.00622-16.

Hussain, S., C. N. Wivagg, P. Szwedziak, F. Wong, K. Schaefer, T. Izore, L. D. Renner, M. J. Holmes, Y. Sun, A. W. Bisson-Filho, S. Walker, A. Amir, J. Lowe and E. C. Garner (2018). "MreB filaments align along greatest principal membrane curvature to orient cell wall synthesis." Elife 7 doi: 10.7554/eLife.32471.

Jakob, U., W. Muse, M. Eser and J. C. Bardwell (1999). "Chaperone activity with a redox switch." Cell 96(3): 341-352.

Kadokura, H. and J. Beckwith (2010). "Mechanisms of oxidative protein folding in the bacterial cell envelope." Antioxid Redox Signal 13(8): 1231-1246 doi: 10.1089/ars.2010.3187.

Kadokura, H., F. Katzen and J. Beckwith (2003). "Protein disulfide bond formation in prokaryotes." Annu Rev Biochem 72: 111-135 doi: 10.1146/annurev.biochem.72.121801.161459.

Kamisetty, H., S. Ovchinnikov and D. Baker (2013). "Assessing the utility of coevolution-based residue-residue contact predictions in a sequence- and structure-rich era." Proc Natl Acad Sci U S A 110(39): 15674-15679 doi: 10.1073/pnas.1314045110.

Kerner, M. J., D. J. Naylor, Y. Ishihama, T. Maier, H. C. Chang, A. P. Stines, C. Georgopoulos, D. Frishman, M. Hayer-Hartl, M. Mann and F. U. Hartl (2005). "Proteome-wide analysis of chaperonin-dependent protein folding in Escherichia coli." Cell 122(2): 209-220 doi: 10.1016/j.cell.2005.05.028.

Koldewey, P., S. Horowitz and J. C. A. Bardwell (2017). "Chaperone-client interactions: Non-specificity engenders multifunctionality." J Biol Chem 292(29): 12010-12017 doi: 10.1074/jbc.R117.796862.

Kopito, R. R. (2000). "Aggresomes, inclusion bodies and protein aggregation." Trends Cell Biol 10(12): 524-530.

Krogh, A., B. Larsson, G. von Heijne and E. L. Sonnhammer (2001). "Predicting transmembrane protein topology with a hidden Markov model: application to complete genomes." J Mol Biol 305(3): 567-580 doi: 10.1006/jmbi.2000.4315.

Kudva, R., P. Tian, F. Pardo-Avila, M. Carroni, R. B. Best, H. D. Bernstein and G. von Heijne (2018). "The shape of the bacterial ribosome exit tunnel affects cotranslational protein folding." Elife 7 doi: 10.7554/eLife.36326.

Kyte, J. and R. F. Doolittle (1982). "A simple method for displaying the hydropathic character of a protein." J Mol Biol 157(1): 105-132 doi: 0022-2836(82)90515-0 [pii].

Laloux, G. and C. Jacobs-Wagner (2014). "How do bacteria localize proteins to the cell pole?" J Cell Sci 127(Pt 1): 11-19 doi: 10.1242/jcs.138628.

Lee, S. F. and L. Davey (2017). "Disulfide Bonds: A Key Modification in Bacterial Extracytoplasmic Proteins." J Dent Res 96(13): 1465-1473 doi: 10.1177/0022034517725059.

Leuenberger, P., S. Ganscha, A. Kahraman, V. Cappelletti, P. J. Boersema, C. von Mering, M. Claassen and P. Picotti (2017). "Cell-wide analysis of protein thermal unfolding reveals determinants of thermostability." Science 355(6327) doi: 10.1126/science.aai7825.

Li, G. W., D. Burkhardt, C. Gross and J. S. Weissman (2014). "Quantifying absolute protein synthesis rates reveals principles underlying allocation of cellular resources." Cell 157(3): 624-635 doi: 10.1016/j.cell.2014.02.033.

Li, N., J. Lv and D. K. Niu (2009). "Low contents of carbon and nitrogen in highly abundant proteins: evidence of selection for the economy of atomic composition." J Mol Evol 68(3): 248-255 doi: 10.1007/s00239-009-9199-4.

Liberman, L., H. C. Berg and V. Sourjik (2004). "Effect of chemoreceptor modification on assembly and activity of the receptor-kinase complex in Escherichia coli." J Bacteriol 186(19): 6643-6646 doi: 10.1128/JB.186.19.6643-6646.2004.

Lu, S., S. B. Fan, B. Yang, Y. X. Li, J. M. Meng, L. Wu, P. Li, K. Zhang, M. J. Zhang, Y. Fu, J. Luo, R. X. Sun, S. M. He and M. Q. Dong (2015). "Mapping native disulfide bonds at a proteome scale." Nat Methods 12(4): 329-331 doi: 10.1038/nmeth.3283.

Luders, S., C. Fallet and E. Franco-Lara (2009). "Proteome analysis of the Escherichia coli heat shock response under steady-state conditions." Proteome Sci 7: 36 doi: 10.1186/1477-5956-7-36.

Maddock, J. R. and L. Shapiro (1993). "Polar location of the chemoreceptor complex in the Escherichia coli cell." Science 259(5102): 1717-1723.

Makino, T., G. Skretas and G. Georgiou (2011). "Strain engineering for improved expression of recombinant proteins in bacteria." Microb Cell Fact 10: 32 doi: 10.1186/1475-2859-10-32.

Malki, A., H. T. Le, S. Milles, R. Kern, T. Caldas, J. Abdallah and G. Richarme (2008). "Solubilization of protein aggregates by the acid stress chaperones HdeA and HdeB." J Biol Chem 283(20): 13679-13687 doi: 10.1074/jbc.M800869200.

Mapa, K., S. Tiwari, V. Kumar, G. G. Jayaraj and S. Maiti (2012). "Information encoded in non-native states drives substrate-chaperone pairing." Structure 20(9): 1562-1573 doi: 10.1016/j.str.2012.06.014.

Marino, J., G. von Heijne and R. Beckmann (2016). "Small protein domains fold inside the ribosome exit tunnel." FEBS Lett 590(5): 655-660 doi: 10.1002/1873-3468.12098.

Martin, J. L. (1995). "Thioredoxin--a fold for all reasons." Structure 3(3): 245-250.

Martinez-Hackert, E. and W. A. Hendrickson (2009). "Promiscuous substrate recognition in folding and assembly activities of the trigger factor chaperone." Cell 138(5): 923-934 doi: 10.1016/j.cell.2009.07.044.

Melo, F., R. Sanchez and A. Sali (2002). "Statistical potentials for fold assessment." Protein Sci 11(2): 430-448 doi: 10.1002/pro.110430.

Meszaros, B., G. Erdos and Z. Dosztanyi (2018). "IUPred2A: context-dependent prediction of protein disorder as a function of redox state and protein binding." Nucleic Acids Res 46(W1): W329-W337 doi: 10.1093/nar/gky384.

Meyer, A. S. and T. A. Baker (2011). "Proteolysis in the Escherichia coli heat shock response: a player at many levels." Curr Opin Microbiol 14(2): 194-199 doi: 10.1016/j.mib.2011.02.001.

Mujacic, M. and F. Baneyx (2006). "Regulation of Escherichia coli hchA, a stress-inducible gene encoding molecular chaperone Hsp31." Mol Microbiol 60(6): 1576-1589 doi: 10.1111/j.1365-2958.2006.05207.x.

Niwa, T., T. Kanamori, T. Ueda and H. Taguchi (2012). "Global analysis of chaperone effects using a reconstituted cell-free translation system." Proc Natl Acad Sci U S A 109(23): 8937-8942 doi: 10.1073/pnas.1201380109.

Niwa, T., B. W. Ying, K. Saito, W. Jin, S. Takada, T. Ueda and H. Taguchi (2009). "Bimodal protein solubility distribution revealed by an aggregation analysis of the entire ensemble of Escherichia coli proteins." Proc Natl Acad Sci U S A 106(11): 4201-4206 doi: 0811922106 [pii] 10.1073/pnas.0811922106.

Oh, E., A. H. Becker, A. Sandikci, D. Huber, R. Chaba, F. Gloge, R. J. Nichols, A. Typas, C. A. Gross, G. Kramer, J. S. Weissman and B. Bukau (2011). "Selective ribosome profiling reveals the cotranslational chaperone action of trigger factor in vivo." Cell 147(6): 1295-1308 doi: 10.1016/j.cell.2011.10.044.

Okuda, S. and H. Tokuda (2011). "Lipoprotein sorting in bacteria." Annu Rev Microbiol 65: 239-259 doi: 10.1146/annurev-micro-090110-102859.

Perrett, S., R. Zahn, G. Stenberg and A. R. Fersht (1997). "Importance of electrostatic interactions in the rapid binding of polypeptides to GroEL." J Mol Biol 269(5): 892-901 doi: 10.1006/jmbi.1997.1081.

Pieper, U., B. M. Webb, G. Q. Dong, D. Schneidman-Duhovny, H. Fan, S. J. Kim, N. Khuri, Y. G. Spill, P. Weinkam, M. Hammel, J. A. Tainer, M. Nilges and A. Sali (2014). "ModBase, a database of annotated comparative protein structure models and associated resources." Nucleic Acids Res 42(Database issue): D336-346 doi: 10.1093/nar/gkt1144.

Piovesan, D., F. Tabaro, I. Micetic, M. Necci, F. Quaglia, C. J. Oldfield, M. C. Aspromonte, N. E. Davey, R. Davidovic, Z. Dosztanyi, A. Elofsson, A. Gasparini, A. Hatos, A. V. Kajava, L. Kalmar, E. Leonardi, T. Lazar, S. Macedo-Ribeiro, M. Macossay-Castillo, A. Meszaros, G. Minervini, N. Murvai, J. Pujols, D. B. Roche, E. Salladini, E. Schad, A. Schramm, B. Szabo, A. Tantos, F. Tonello, K. D. Tsirigos, N. Veljkovic, S. Ventura, W. Vranken, P. Warholm, V. N. Uversky, A. K. Dunker, S. Longhi, P. Tompa and S. C. Tosatto (2017). "DisProt 7.0: a major update of the database of disordered proteins." Nucleic Acids Res 45(D1): D1123-D1124 doi: 10.1093/nar/gkw1279.

Piovesan, D., F. Tabaro, L. Paladin, M. Necci, I. Micetic, C. Camilloni, N. Davey, Z. Dosztanyi, B. Meszaros, A. M. Monzon, G. Parisi, E. Schad, P. Sormanni, P. Tompa, M. Vendruscolo, W. F. Vranken and S. C. E. Tosatto (2018). "MobiDB 3.0: more annotations for intrinsic disorder, conformational diversity and interactions in proteins." Nucleic Acids Res 46(D1): D471-D476 doi: 10.1093/nar/gkx1071.

Plaxco, K. W., K. T. Simons and D. Baker (1998). "Contact order, transition state placement and the refolding rates of single domain proteins." J Mol Biol 277(4): 985-994 doi: 10.1006/jmbi.1998.1645.

Powers, E. T., D. L. Powers and L. M. Gierasch (2012). "FoldEco: a model for proteostasis in E. coli." Cell Rep 1(3): 265-276 doi: 10.1016/j.celrep.2012.02.011.

Prinz, W. A., F. Aslund, A. Holmgren and J. Beckwith (1997). "The role of the thioredoxin and glutaredoxin pathways in reducing protein disulfide bonds in the Escherichia coli cytoplasm." J Biol Chem 272(25): 15661-15667.

Quan, S., P. Koldewey, T. Tapley, N. Kirsch, K. M. Ruane, J. Pfizenmaier, R. Shi, S. Hofmann, L. Foit, G. Ren, U. Jakob, Z. Xu, M. Cygler and J. C. Bardwell (2011). "Genetic selection designed to stabilize proteins uncovers a chaperone called Spy." Nat Struct Mol Biol 18(3): 262-269 doi: 10.1038/nsmb.2016.

Raimondi, D., G. Orlando, R. Pancsa, T. Khan and W. F. Vranken (2017). "Exploring the Sequence-based Prediction of Folding Initiation Sites in Proteins." Sci Rep 7(1): 8826 doi: 10.1038/s41598-017-08366-3.

Randall, L. L., S. J. Hardy, T. B. Topping, V. F. Smith, J. E. Bruce and R. D. Smith (1998). "The interaction between the chaperone SecB and its ligands: evidence for multiple subsites for binding." Protein Sci 7(11): 2384-2390 doi: 10.1002/pro.5560071115.

Richter, K., M. Haslbeck and J. Buchner (2010). "The heat shock response: life on the verge of death." Mol Cell 40(2): 253-266 doi: 10.1016/j.molcel.2010.10.006.

Rose, P. W., A. Prlic, A. Altunkaya, C. Bi, A. R. Bradley, C. H. Christie, L. D. Costanzo, J. M. Duarte, S. Dutta, Z. Feng, R. K. Green, D. S. Goodsell, B. Hudson, T. Kalro, R. Lowe, E. Peisach, C. Randle, A. S. Rose, C. Shao, Y. P. Tao, Y. Valasatava, M. Voigt, J. D. Westbrook, J. Woo, H. Yang, J. Y. Young, C. Zardecki, H. M. Berman and S. K. Burley (2017). "The RCSB protein data bank: integrative view of protein, gene and 3D structural information." Nucleic Acids Res 45(D1): D271-D281 doi: 10.1093/nar/gkw1000.

Ross, C. A. and M. A. Poirier (2004). "Protein aggregation and neurodegenerative disease." Nat Med 10 Suppl: S10-17 doi: 10.1038/nm1066.

Roy, A., A. Kucukural and Y. Zhang (2010). "I-TASSER: a unified platform for automated protein structure and function prediction." Nat Protoc 5(4): 725-738 doi: 10.1038/nprot.2010.5.

Rudner, D. Z. and R. Losick (2010). "Protein subcellular localization in bacteria." Cold Spring Harb Perspect Biol 2(4): a000307 doi: 10.1101/cshperspect.a000307.

Saio, T., X. Guan, P. Rossi, A. Economou and C. G. Kalodimos (2014). "Structural basis for protein antiaggregation activity of the trigger factor chaperone." Science 344(6184): 1250494 doi: 10.1126/science.1250494.

Santra, M., D. W. Farrell and K. A. Dill (2017). "Bacterial proteostasis balances energy and chaperone utilization efficiently." Proc Natl Acad Sci U S A 114(13): E2654-E2661 doi: 10.1073/pnas.1620646114.

Schibich, D., F. Gloge, I. Pohner, P. Bjorkholm, R. C. Wade, G. von Heijne, B. Bukau and G. Kramer (2016). "Global profiling of SRP interaction with nascent polypeptides." Nature 536(7615): 219-223 doi: 10.1038/nature19070.

Schmidt, A., K. Kochanowski, S. Vedelaar, E. Ahrne, B. Volkmer, L. Callipo, K. Knoops, M. Bauer, R. Aebersold and M. Heinemann (2016). "The quantitative and condition-dependent Escherichia coli proteome." Nat Biotechnol 34(1): 104-110 doi: 10.1038/nbt.3418.

Shapiro, L., H. H. McAdams and R. Losick (2009). "Why and how bacteria localize proteins." Science 326(5957): 1225-1228 doi: 10.1126/science.1175685.

Shi, Y., J. Zhou, D. Arndt, D. S. Wishart and G. Lin (2008). "Protein contact order prediction from primary sequences." BMC Bioinformatics 9: 255 doi: 10.1186/1471-2105-9-255.

Shih, Y. L. and M. Zheng (2013). "Spatial control of the cell division site by the Min system in Escherichia coli." Environ Microbiol 15(12): 3229-3239 doi: 10.1111/1462-2920.12119.

Tomoyasu, T., A. Mogk, H. Langen, P. Goloubinoff and B. Bukau (2001). "Genetic dissection of the roles of chaperones and proteases in protein folding and degradation in the Escherichia coli cytosol." Mol Microbiol 40(2): 397-413.

Tsamardinos, I., E. Greasidou and G. Borboudakis (2018). "Bootstrapping the out-of-sample predictions for efficient and accurate cross-validation." Mach Learn 107(12): 1895-1922 doi: 10.1007/s10994-018-5714-4.

Tsirigotaki, A., K. E. Chatzi, M. Koukaki, J. De Geyter, A. G. Portaliou, G. Orfanoudaki, M. F. Sardis, M. B. Trelle, T. J. D. Jorgensen, S. Karamanou and A. Economou (2018). "Long-Lived Folding Intermediates Predominate the Targeting-Competent Secretome." Structure 26(5): 695-707 e695 doi: 10.1016/j.str.2018.03.006.

Tsirigotaki, A., J. De Geyter, N. Sostaric, A. Economou and S. Karamanou (2017). "Protein export through the bacterial Sec pathway." Nat Rev Microbiol 15(1): 21-36 doi: 10.1038/nrmicro.2016.161.

Tsolis, K. C., E. P. Tsare, G. Orfanoudaki, T. Busche, K. Kanaki, R. Ramakrishnan, F. Rousseau, J. Schymkowitz, C. Ruckert, J. Kalinowski, J. Anne, S. Karamanou, M. I. Klapa and A. Economou (2018). "Comprehensive subcellular topologies of polypeptides in Streptomyces." Microb Cell Fact 17(1): 43 doi: 10.1186/s12934-018-0892-0.

Webb, B. and A. Sali (2016). "Comparative Protein Structure Modeling Using MODELLER." Curr Protoc Bioinformatics 54: 5 6 1-5 6 37 doi: 10.1002/cpbi.3.

Wilson, D., R. Pethica, Y. Zhou, C. Talbot, C. Vogel, M. Madera, C. Chothia and J. Gough (2009). "SUPERFAMILY--sophisticated comparative genomics, data mining, visualization and phylogeny." Nucleic Acids Res 37(Database issue): D380-386 doi: 10.1093/nar/gkn762.

Wu, S. and Y. Zhang (2007). "LOMETS: a local meta-threading-server for protein structure prediction." Nucleic Acids Res 35(10): 3375-3382 doi: 10.1093/nar/gkm251.

Xu, D. and Y. Zhang (2013). "Ab Initio structure prediction for Escherichia coli: towards genome-wide protein structure modeling and fold assignment." Sci Rep 3: 1895 doi: 10.1038/srep01895.
